# The role of antisense long noncoding RNA in small RNA-triggered gene activation

**DOI:** 10.1261/rna.043968.113

**Published:** 2014-12

**Authors:** Xizhe Zhang, Haitang Li, John C. Burnett, John J. Rossi

**Affiliations:** 1 Irell and Manella Graduate School of Biological Sciences, Beckman Research Institute at the City of Hope, Duarte, California 91010, USA; 2 Department of Molecular and Cellular Biology, Beckman Research Institute at the City of Hope, Duarte, California 91010, USA

**Keywords:** lncRNA, shRNA, transcriptional gene activation, TGA, RNA-FISH, Argonaute 2

## Abstract

Long noncoding RNAs (lncRNAs) are known to regulate neighboring protein-coding genes by directing chromatin remodeling complexes, imprinting, and X-chromosome inactivation. In this study, we explore the function of lncRNAs in small RNA-triggered transcriptional gene activation (TGA), a process in which microRNAs (miRNAs) or small interfering RNAs (siRNAs) associated with Argonaute (Ago) proteins induce chromatin remodeling and gene activation at promoters with sequence complementarity. We designed a model system with different lncRNA and chromatin environments to elucidate the molecular mechanisms required for mammalian TGA. Using RNA-fluorescence in situ hybridization (FISH) and rapid amplification of cDNA ends (RACE)-PCR, we demonstrated that small RNA-triggered TGA occurs at sites where antisense lncRNAs are transcribed through the reporter gene and promoter. Small RNA-induced TGA coincided with the enrichment of Ago2 at the promoter region, but Ago2-mediated cleavage of antisense lncRNAs was not observed. Moreover, we examined the allele-specific effects of lncRNAs through a Cre-induced inversion of a poly(A) sequence that was designed to block the transcription of antisense lncRNAs through the reporter gene region in an inducible and reversible manner. Termination of nascent antisense lncRNAs abrogated gene activation triggered by small RNAs, and only allele-specific *cis*-acting antisense lncRNAs, but not *trans-*acting lncRNAs, were capable of rescuing TGA. Hence, this model revealed that antisense lncRNAs can mediate TGA in *cis* and not in *trans,* serving as a molecular scaffold for a small RNA–Ago2 complex and chromatin remodeling.

## INTRODUCTION

It is well known that eukaryotic genomes are pervasively transcribed in both sense and antisense directions, producing abundant levels of noncoding RNAs (ncRNAs) (Cheng et al. 2005; Katayama et al. 2005; Zhang et al. 2006). Long ncRNAs (lncRNAs), ranging from ≈200 nt to several kilobases long, are found to be involved in numerous normal cellular processes and disease states. LncRNAs can function in *trans* or in *cis*, which is a critical factor in understanding the functions of a particular lncRNA within the entire transcriptome (for review, see Guil and Esteller 2012; Guttman and Rinn 2012). Numerous examples of *trans*-acting lncRNAs have been described (Khalil et al. 2009; Orom et al. 2010; Guttman et al. 2011), including the *trans*-lncRNA *HOTAIR* which directs chromatin modulation complexes PCR2 to the *HOXD* locus far away from the region of its transcription (Gupta et al. 2010) and serves as a molecular scaffold for histone-modifying complexes (Tsai et al. 2010). On the other hand, *cis*-lncRNAs, such as *Xist* in X-chromosome inactivation (for review, see Lee 2009) and *Air* in imprinting (Nagano et al. 2008), affect gene expression in allele-specific mode.

In addition to the examples given above, antisense lncRNAs have been associated with the regulation of transcriptional gene activation (TGA) triggered by small RNAs (Schwartz et al. 2008; Chu et al. 2010; Yue et al. 2010; Matsui et al. 2013). TGA refers to the induced or enhanced activation of a specific gene that is mediated by small double-stranded RNAs (dsRNA) complementary to an antisense lncRNA from that promoter region (Janowski et al. 2007). In mammalian cells, this process requires Argonaute 2 (Ago2) and is associated with epigenetic activation of the targeted promoter (Li et al. 2006; Janowski et al. 2007; Morris et al. 2008). The molecular details about the role of lncRNAs in TGA are unclear, and it is important to identify whether they act via a *cis*- or *trans*-mechanism in order to understand the generalities of TGA for biological and therapeutic applications.

In a *cis*-acting model for TGA, the siRNA–Ago2 complex is directed to the genomic promoter region by sequence homology between a promoter-associated lncRNA and the guide siRNA strand (Chu et al. 2010; Matsui et al. 2013). The *cis*-acting lncRNA interacts with the siRNA–Ago2 complex inside the nucleus at the genomic locus (Gagnon and Corey 2012). In this model, the lncRNA is tethered to the promoter region, thereby serving as a scaffold for gene activation by complementary small RNAs and chromatin-modifying factors (Matsui et al. 2013).

While the *cis*-acting model TGA requires the recruitment of a siRNA–Ago2 complex to the genome, similar observations of small RNA gene activation have resulted from off-target post-transcriptional gene silencing (PTGS). *Trans*-acting lncRNAs that repress gene transcription of homology-containing loci can be targeted by siRNAs, and the PTGS-induced knockdown of the lncRNAs can lead to derepression of gene transcription (Weinberg and Morris 2013). For instance, despite the initial observation that dsRNAs could induce gene reactivation of the p21 gene in human cells (Li et al. 2006), a later study revealed that these effects were instead the PTGS knockdown of a *trans*-acting suppressive antisense transcript (Morris et al. 2008). In this scenario, the lncRNAs function as a *tran*s-acting factor to repress the gene expression.

Despite these and other demonstrations of mammalian TGA (Place et al. 2008; Matilainen et al. 2010; Matsui et al. 2010; Voutila et al. 2012; Reebye et al. 2013a,b), the molecular mechanisms of the process are still not well understood, including the details about the role of lncRNAs in TGA and whether they act in an allele-specific *cis*-mode or a *trans*-mode. In this study, we devised a TGA model based on *CMV-EGFP* reporter gene system to investigate the factors required for TGA. By targeting a region within the *CMV* promoter with shRNAs, we demonstrated that the induction of TGA depends on the chromatin environment of the reporter gene and the transcription of antisense lncRNAs through the promoter. Transcriptional inhibition of such antisense lncRNAs abrogated TGA. Our model supports a TGA mechanism in which lncRNAs act in *cis*, serving as the scaffold to recruit small RNA-programmed Ago2 complexes to the target promoter region to induce gene activation. While this process requires Ago2, it is independent of any cleavage activity, indicating that the ensuing gene activation is unlikely the consequence of Ago2-mediated lncRNA knockdown.

## RESULTS

### Experimental design for analyzing TGA in mammalian cells

**FIGURE 1.**
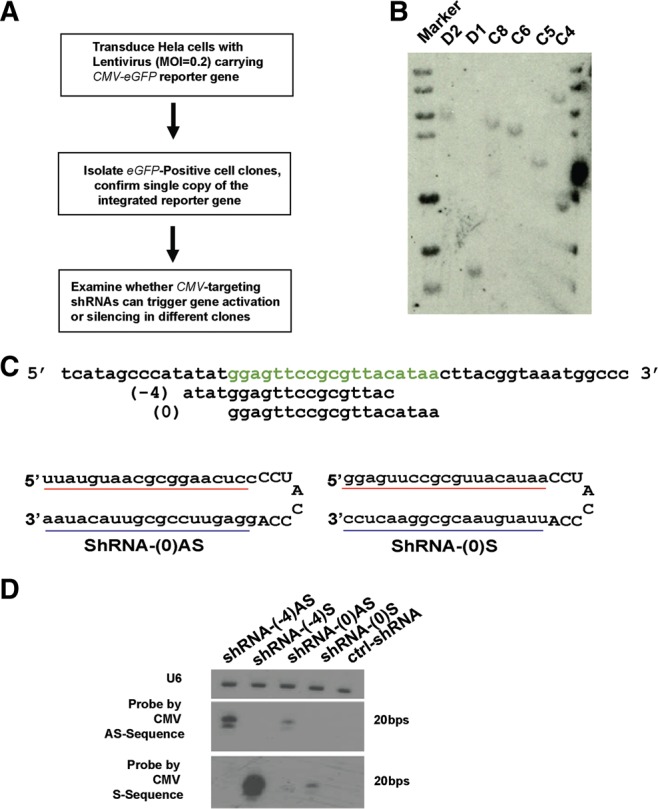
A cell model to evaluate the effect of local chromatin environment on shRNA-mediated TGA. (*A*) Experimental design for analyzing TGA in HeLa clones. (*B*) Southern blot to analyze if isolated cell clones, such as D2, D1, C8, C6, C5, and C4, have one copy of the *CMV-EGFP* reporter gene integrated into the genome. All clones were isolated from the same transduced HeLa cell population. (*C*) The design of shRNAs targeting the *CMV* promoter. The sequence in green is denoted as site 0, which corresponds to the region −531 to −513 bp upstream of the transcriptional starting site (TSS). The numbers in parentheses denote the *CMV* promoter region targeted by each shRNA. The positions of the *bottom* (guide) and *upper* (passenger) strands in shRNA-(0)S (*lower right*) are reversed in shRNA(0)AS (*lower left*). (*D*) Northern blot to analyze preferential selection of the *bottom* shRNA strands as the guide strands. HeLa cells were transfected by the plasmids expressing these shRNAs (see Materials and Methods for details). An irrelevant shRNA is used as the negative control (ctrl-shRNA). U6 RNA was probed for the loading control. The images were taken from the same membrane hybridized with different probes.

In this study, we developed a model to elucidate the mechanisms and necessary factors for small RNA-triggered TGA in mammalian cells. This system is designed for testing shRNA-induced TGA for the same promoter across a range of different integration sites to vary the chromatin environment and antisense transcription (Fig. 1A). First, HeLa cells were transduced with recombinant lentivirus for the integration of a reporter gene cassette in a semirandom fashion across the genome (Schroder et al. 2002). The lentiviral vector is composed of human cytomegalovirus immediate early promoter (*CMV*) and downstream enhanced green fluorescent protein (*eGFP*) gene. The *CMV* promoter was chosen over other commonly used human promoters (e.g., *EF1*α, *PGK*, and *ubiquitin C*), since the *CMV* promoter does not occur in the human genome and thus any possible effects would not be convoluted by the same endogenous promoters. Also, by examining cells with a single integrated copy of the *CMV-EGFP* lentivirus, we could analyze the effects of TGA at a single promoter rather than the endogenous promoters at both loci.

HeLa cells were transduced with the *CMV-EGFP* lentivirus a low multiplicity of infection (MOI = 0.2) to minimize the likelihood of multiple infections. Next, we isolated individual cell clones, confirmed single-integration of the reporter gene by Southern blot (Fig. 1B), and mapped the integration sites by inverse PCR (Supplemental Table S1). We designed a library of short hairpin RNAs (shRNAs) against the *CMV* promoter to screen for those that enhance eGFP expression as a potential indicator of TGA. The transfected shRNAs are precursors to siRNAs that are processed by Dicer into ≈21 nt RNA duplexes (Paddison et al. 2002). To screen for a TGA response, we first screened shRNAs at ≈100-bp intervals across the entire *CMV* promoter and identified one TGA-inducing shRNA that spans positions −531 to −513 upstream of the transcriptional start site (TSS) (Fig. 1C; Supplemental Table S2). This shRNA was denoted as shRNA-(0), and additional shRNAs were designed by shifting the target site upstream of or downstream from shRNA-(0). For instance, the target site of shRNA-(-4) is 4-nt upstream relative to the site targeted by shRNA-(0) (Fig. 1C). For each target site, two shRNAs were designed and denoted as “AS” or “S,” by exchanging the placement of the upper and lower strands with respect to the shRNA hairpin loop (Fig. 1C, bottom left and bottom right). Using shRNAs instead of siRNAs in this study is based on the observation that the bottom strand (3′ arm) of the shRNAs is preferentially selected as the guide strand, which is informative to identify the polarity of the endogenous RNA targets (Fig. 1D). Thus, the “AS” in the shRNA name implies that it is more likely to target on antisense sequences, whereas “S” refers to it preferentially targeting the sense sequences.

### TGA is dependent on shRNA sequence and chromatin environment of the promoter

**FIGURE 2.**
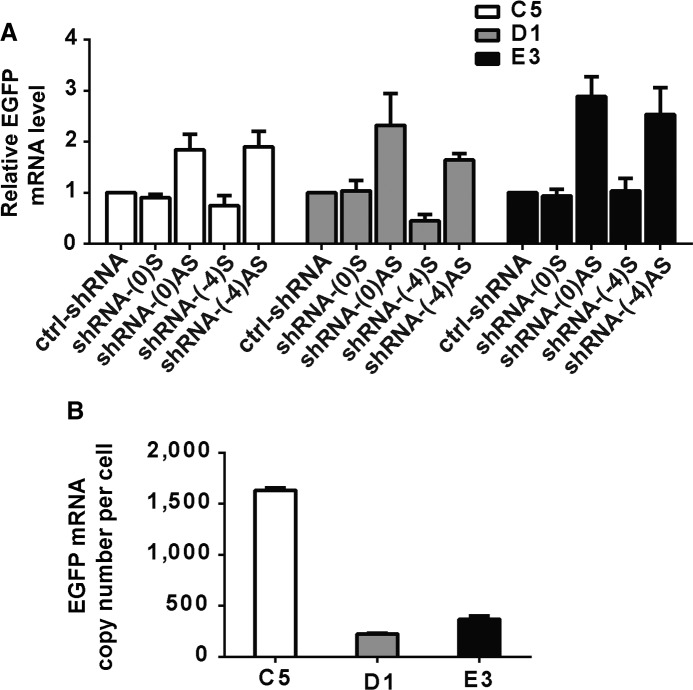
*CMV*-targeting shRNAs trigger TGA in the clones C5, D1, and E3. (*A*) Relative expression level of eGFP in *CMV-EGFP* clones C5, D1, and E3, as determined by RT-PCR and normalized to housekeeping gene *HPRT1*. In this study, all experiments were repeated three times independently and data are represented as mean ± SD. (*B*) The constitutive eGFP expression represented as the copy numbers of mRNA molecules per cell in three clones, D1, C5, and E3, as determined by RT-PCR.

A total of 16 shRNAs tiled across the *CMV* promoter shifted by 1 bp were screened for the ability to trigger TGA. Two such shRNAs, termed shRNA-(-4)AS and shRNA-(0)AS, enhanced eGFP expression in one specific HeLa clone named D1 (Supplemental Fig. S1A). We further analyzed these two shRNAs (shRNA-(0)AS and shRNA-(-4)AS) and their mirrored partners (shRNA-(0)S and shRNA-(-4)S) to test their ability to trigger gene activation in other isolated HeLa clones. We found that both shRNA-(0)AS and shRNA-(-4)AS triggered increased eGFP expression in three clones (C5, D1, and E3), whereas it is not the case in the other *CMV-EGFP* clones (Fig. 2A; Supplemental Fig. S1B). The activation of EGFP expression was also detected at the protein level (Supplemental Fig. S1C). These results support the hypothesis that shRNA-mediated TGA is not merely a consequence of the promoter sequence, but is also affected by the genomic loci and/or the local chromatin environment of the promoter. No strong correlation was observed between the initial eGFP expression level and the ability to trigger TGA in the 10 clones tested (Fig. 2A,B; Supplemental Fig. S1B,D). However, a low or moderate expression level of *CMV*-driven gene expression appears to be necessary, but not sufficient, for shRNA-induced TGA, as the clones with the highest eGFP expression levels (C3 and D2) were not further enhanced by TGA (Supplemental Fig. S1B,D).

### TGA is mediated by Ago2 at the chromatin and epigenetic changes

**FIGURE 3.**
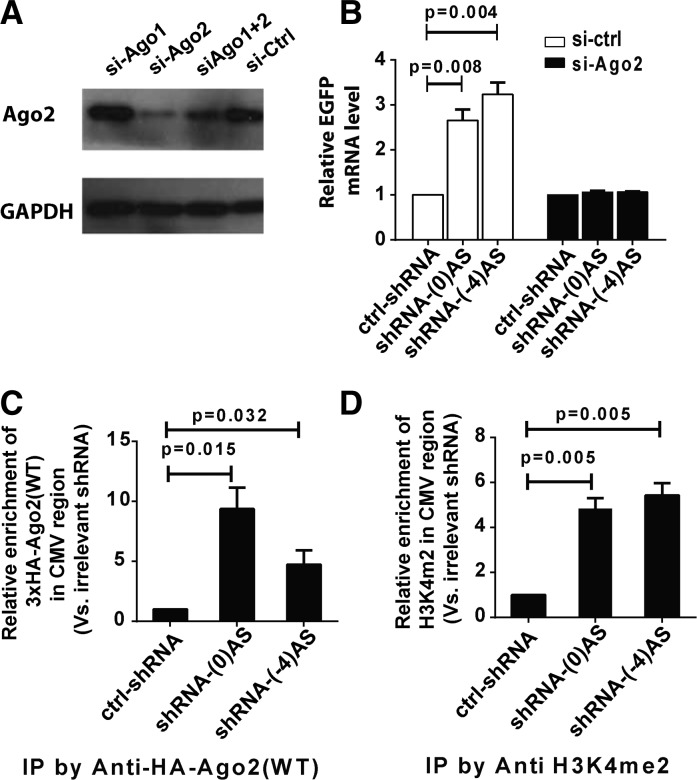
shRNA-triggered TGA is associated with recruitment of Ago2 and enrichment of gene activation marker into the *CMV* region. (*A*) Western blot of Ago2 protein after knockdown using siRNAs against Ago1, Ago2, the combination of both, or an irrelevant siRNA. (*B*) Quantitative RT-PCR to detect TGA response in normal C5 clones (white bars) or C5 clones with depleted levels of endogenous Ago2 (black bars). Anti-Ago2 siRNAs and plasmids expressing TGA-inducing shRNAs were cotransfected into cells. The RNA samples were extracted 4 d post-transfection for analysis. (*C*) ChIP analysis for the enrichment of Ago2 at the *CMV* promoter region after shRNA-mediated gene activation in the C5 clone. Plasmids expressing 3xHA-Ago2 driven by the ubiquitin promoter were cotransfected with shRNA-(0)AS, shRNA-(-4)AS, and an irrelevant shRNA (ctrl-shRNA) into cells. ChIP results were represented as the fold of enrichment relative to an irrelevant shRNA. Statistical analysis in this study was performed using a two-tailed *t*-test. (*D*) ChIP analysis for H3K4me2 at the *CMV* region after shRNA-mediated gene activation in C5 clone.

Previous studies have established the role of Ago2 for small RNA-mediated TGA (Janowski et al. 2007; Chu et al. 2010). To test whether Ago2 is involved in shRNA-triggered gene activation in our system, we first examined the effects of TGA in Ago2-depleted conditions. In clone C5, knockdown of Ago2 attenuated the levels of TGA induction by the promoter-targeting shRNAs (Fig. 3A,B). The requirement of Ago2 for TGA was reproduced in clone D1 (Supplemental Fig. S2A). Next, we asked whether Ago2 was localized at the *CMV* promoter in clones that undergo gene activation by shRNA-(0) or shRNA-(-4). We transiently coexpressed the shRNAs with 3xHA-Ago2 protein or 3xHA-Ago2 mutant, whose catalytic center is compromised (Liu et al. 2004), in the C5 clone (Supplemental Fig. S2B–D). Ectopically expressed wild-type and mutant HA-tagged Ago2 proteins were functional and able to incorporate processed shRNAs, as determined by immunoprecipitation followed by Northern blot analysis (Supplemental Fig. S2D). Consistent with previous studies (Chu et al. 2010; Matsui et al. 2013), Ago2 was enriched in the *CMV* promoter region during shRNA-mediated TGA as determined by chromatin immunoprecipitation (ChIP) (Fig. 3C), with no apparent enrichment in the control GAPDH promoter genomic locus (Supplemental Fig. S2E). Additionally, ChIP revealed that the Ago2 mutant, which contains Q633R and H634P mutations in the catalytic center, was enriched in the *CMV* promoter region in clones treated with TGA-inducing shRNAs (Supplemental Fig. S2F). Finally, we observed increased dimethylation of H3K4 at the *CMV* promoter in TGA-induced cells, a hallmark for promoter activation (Fig. 3D; Janowski et al. 2007).

### Levels of promoter-associated antisense lncRNAs do not correlate with shRNA-mediated TGA

For the shRNAs that induced a TGA phenotype (shRNA-(-4)AS and shRNA-(0)AS), the guide strands of the shRNAs were designed to target an antisense sequence at the *CMV* promoter. Thus we performed strand-specific RT-PCR to detect transcripts in the antisense orientation of the *CMV-EGFP-WPRE* lentiviral vector. Indeed, antisense lncRNAs spanning the promoter region were identified among all integration sites by RT-PCR (Supplemental Fig. S3A).

**FIGURE 4.**
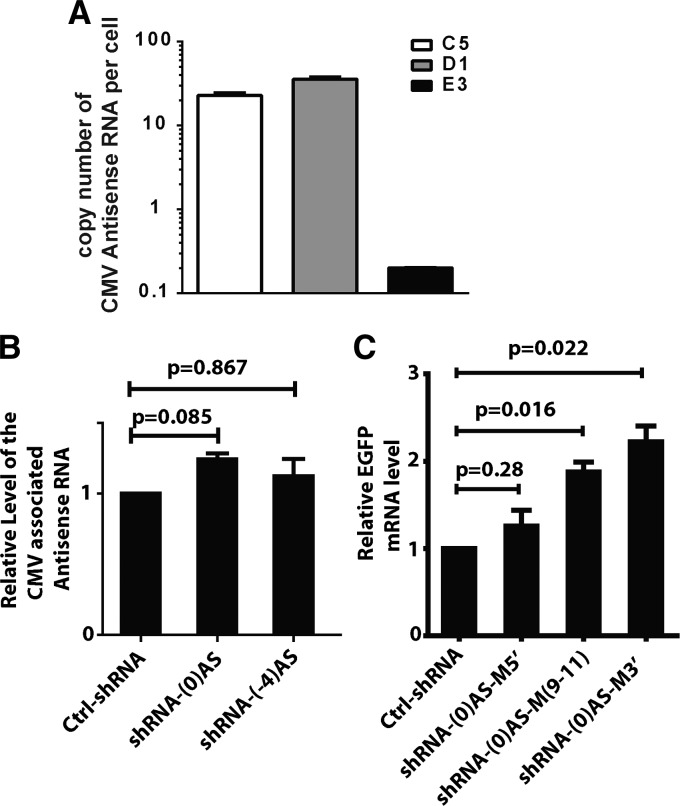
TGA is independent of the cleavage activity of Ago2 and the levels of steady-state promoter-associated RNAs do not correlate to TGA. (*A*) Strand-specific qRT-PCR to detect promoter-associated antisense RNA in clones C5, D1, and E3. Standard curves were generated from genomic DNA standards of the same gene. Data are normalized as cDNA copies per cell, with the assumption that 1 μg cDNA corresponds to 10^5^ cells. (*B*) Strand-specific qRT-PCR for the antisense RNA from clone C5 4 d after treatment with irrelevant shRNA (ctrl-shRNA) or the TGA-inducing shRNAs (shRNA-(0)AS and shRNA-(-4)). Statistical analysis in this study was performed using a two-tailed *t*-test. (*C*) Strand-specific qRT-PCR for the sense *eGFP* mRNA from clone C5 4 d after treatment of irrelevant shRNA (ctrl-shRNA) or three mutated variants of shRNA-(0). The strand of shRNA-(0)AS is mutated at nucleotides 1–5 (shRNA-(0)AS-M5′), nucleotides 9–11 (shRNA-(0)AS-M(9-11)), or nucleotides 15–19 (shRNA-(0)AS-M3′). Sequences for the shRNAs are provided in Supplemental Table S2.

We next examined the relationship between the levels of promoter-associated antisense RNA and the occurrence of TGA in various *CMV-EGFP* clones using strand-specific qRT-PCR with primers within the *CMV* region. The levels of antisense RNA were much lower than the *EGFP* mRNA, at the range from 0.1 to 40 copies per cell (Fig. 4A; Supplemental Fig. S3B). High levels of promoter-associated antisense transcripts were detected in clones C5 and D1 (about 22 and 36 copies per cell, respectively), but levels were significantly lower in clone E3 (about 0.2 copy per cell) (Fig. 4A). Nonetheless, TGA was triggered in all three clones (Fig. 2A; Supplemental Fig. S1B). Moreover, although TGA was not observed in clones C4, C6, and C8, these clones possess comparable levels of antisense transcripts as C5 and D1, indicating that a steady-state level of antisense transcripts are not sufficient to indicate the occurrence of TGA (Fig. 4A; Supplemental Fig. S3B).

### TGA does not require the shRNA-induced cleavage of antisense lncRNAs

Our experimental system suggests a model for TGA that requires Ago2 (Fig. 3B,C), but does not appear to require a mechanism involving the Ago2-mediated cleavage of antisense transcripts, since the noncleaving catalytic mutant Ago2 incorporates TGA-inducing shRNAs (Supplemental Fig. S2D) and the mutant Ago2 is enriched at the TGA-induced promoter (Supplemental Fig. S2F). These observations are consistent with a mechanism distinct from post-transcriptional gene silencing (PTGS), in which shRNAs induce Ago2-mediated cleavage of target transcripts. Thus, we aimed to examine whether promoter-associated antisense transcripts undergo shRNA-guided cleavage during TGA.

We designed a series of experiments to determine if the antisense transcript undergoes shRNA-mediated cleavage, which would support a PTGS mechanism. First, we observed that shRNA-(0)AS and shRNA(-4)AS did not significantly alter the level of the promoter-associated antisense transcripts spanning the *CMV* region (Fig. 4B), despite each of these inducing TGA of the *CMV* promoter (Fig. 2A). Next, we hypothesized that mutations to shRNA might preserve any potential Ago2-mediated TGA effects, but would preclude Ago2-mediated cleavage of the antisense transcripts under a PTGS mechanism that requires perfect or near-perfect homology. Therefore, we created three variants of shRNA-(0)AS with mutations at three different regions of the guide strand: positions 1–5 in the 5′ terminus, positions 9–11 in the middle, and positions 15–19 in the 3′ terminus, named shRNA-(0)AS-M5′, shRNA-(0)AS-M(9-11), and shRNA-(0)AS-M3′, respectively (Supplemental Table S2). Transfection of the mutant shRNAs in C5 and D1 clones revealed that mutation within the center or on 3′ terminus of the shRNA did not affect TGA, though mutation to the 5′ terminus of the guide strand abated TGA (Fig. 4C; Supplemental Fig. S3C). Given that mutation to the 5′ terminus of the guide strand would destroy the seed sequences, it is likely that a seed-type pairing is involved in TGA. The fact that shRNA-(0)AS-M5′ was unable to induce TGA is consistent with such a mechanism. However, both shRNA-(0)AS-M(9-11) and shRNA-(0)AS-M3′ significantly activated eGFP expression, consistent with a TGA mechanism. Collectively, these results indicate that the requirement for Ago2 protein in shRNA-mediated TGA is not related to its cleavage activity on the promoter-spanning antisense lncRNAs.

### Analysis of the origin and termination sites for antisense lncRNAs

**FIGURE 5.**
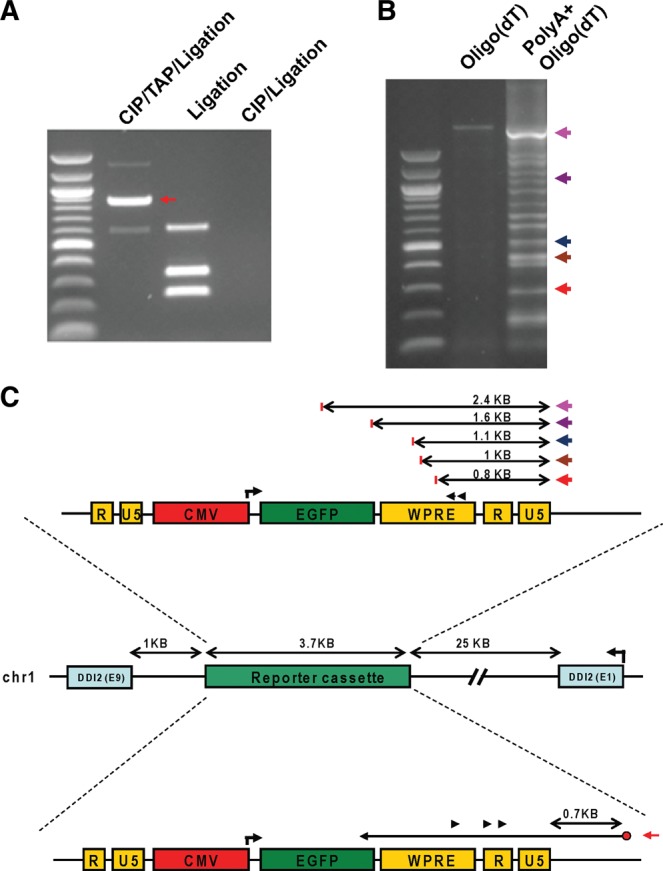
5′ RACE and 3′ RACE indicate antisense lncRNAs in the integration site in the C5 clone. (*A*) 5′ RACE PCR to detect the transcription of antisense lncRNA C5-AS1 into the lentiviral integration site of clone C5. C5-AS1 originates from the adjacent genomic region 0.7 kb downstream from the 3′ terminus of the integrated cassette. CIP/TAP/Ligation (*left* lane) total RNAs were treated by calf-intestinal alkaline phosphatase (CIP), tobacco acid pyrophosphatase (TAP), and ligated to RNA adaptors before analysis by nested PCR. Only lncRNAs with an intact 5′ cap structure can be detected by this method. Ligation (*middle* lane) total RNAs were treated by CIP and TAP sequentially before they were ligated to RNA adaptor and subjected to nested PCR analysis. The lncRNAs with monophosphate can be detected by this method. CIP/Ligation (*right* lane) total RNAs were treated by CIP, ligated to RNA adaptors, and analyzed by nested PCR. This lane shows the general background of 5′ RACE. The gel bands indicate lengths of nested PCR products, not the absolute lengths of antisense RNA. (*B*) 3′ RACE PCR to detect the antisense lncRNAs that are transcribed into the reporter gene cassettes. The total RNAs were reverse transcribed by the adaptor-oligo(dT) primer (*left* lane). The total RNAs were first tailed with poly(A) sequence before reverse transcription (*right* lane). The RT products were analyzed by nested PCR and sequencing. Multiple termini were detected, as indicated by the colored arrows. The bands on the gel indicate lengths of nested PCR products, not the absolute lengths of antisense RNA. (*C*) Schematic diagram shows that a single 5′ terminus (5′ RACE, *bottom*) and multiple 3′ termini (3′ RACE, *upper*) and were detected for the antisense lncRNAs that are transcribed into the integration site in C5 clone. In the *upper* section, the colored arrows represent the 3′ termini of the antisense lncRNAs that were mapped within the reporter cassette. For each of the five lncRNA 3′ termini detected in *B*, the distance from the 3′ termini of the lncRNA to the 3′ termini of the reporter cassette are indicated. In the *lower* section, the 5′ terminus of C5-AS1 was mapped within the genomic region downstream from the reporter gene cassette. The distance from the antisense lncRNA starting site to the 3′ terminus of the reporter cassette is indicated.

The antisense RNAs spanning the *CMV* promoter can originate from the cryptic promoters within the vector or from the neighboring genomic region. We performed 5′-RACE (rapid amplification of cDNA ends) PCR and 3′-RACE PCR to identify the origin and termination of these antisense lncRNAs. The primers for the reverse transcription and nested PCR were localized within the woodchuck hepatitis virus post-transcriptional regulatory element (WPRE) and U5 and U3 regions of the vector sequences. Using 5′-RACE PCR, we detected antisense transcripts originating from the neighboring genomic region, which were transcribed into the integrated reporter gene cassette for C5, D1, and E3 clones (Fig. 5A; Supplemental Figs. S4A, S5A). In C5 cells, at least one antisense lncRNA (named as C5-AS1) with the intact 5′ cap structure was detected that transverses the integration site (Fig. 5A,C). The C5-AS1 lncRNA originated from the genomic region (gene *DDI2*, Accession: NM_032341.4), which is 700-nt away from the 3′ terminus of the integrated vector (Fig. 5C; Supplemental Text S1). Using 3′-RACE, we identified multiple different 3′ termini for the antisense lncRNAs localized within the 3′ terminus of the integrated vector (Fig. 5B,C; Supplemental Text S2). These 3′ termini are not tailed by poly(A) suggesting that they are generated from the degraded products of antisense lncRNAs.

In D1 cells, antisense lncRNAs with a 5′-monophosphate group were identified with a unique 5′ end, suggesting that these were splicing or degradation products from a larger RNA precursor (Supplemental Fig. S4A,B and Text S3). In E3 cells, no starting site for the antisense lncRNA was identified by 5′-RACE. However, the existence of an antisense lncRNA that was transcribed into the integration site was confirmed by strand-specific PCR with primers within the neighboring vector sequence and genomic sequence (Supplemental Fig. S5A). Additionally, 3′-RACE revealed multiple termini of the antisense lncRNAs in clone C5 (Supplemental Fig. S5B,C and Text S4). The multiple termination sites detected by 3′-RACE for the antisense lncRNAs suggest that these lncRNAs are unstable once they are transcribed. Taken together, these results indicate that antisense transcripts originate from within the genomic regions and traverse the vector sequences in clones C5, D1, and E3, consistent with the hypothesis that shRNA-(-4)AS and shRNA-(0)AS target antisense lncRNAs.

### RNA-FISH reveals a single antisense lncRNA localized in the nucleus

The 5′-RACE and 3′-RACE experiments confirmed that antisense lncRNAs originated from within genome and traversed the *CMV-EGFP* lentiviral sites of integration. However, it is not clear if the detected lncRNAs represented a steady-state accumulation of transcripts throughout the cell, or if they were nascent transcripts that remain tethered to the site of transcription. This distinction is important in understanding whether the antisense lncRNA functions in a *trans-* or *cis*-mode. Therefore, we applied RNA-fluorescence in situ hybridization (RNA-FISH) to visualize the cellular localization and the relative abundance of lncRNAs at single-molecule and single-cell resolution, using a previously described technique (Khalil et al. 2009; Raj and Tyagi 2010; Kretz et al. 2013; Maamar et al. 2013). Using Stellaris FISH Probe Designer (Biosearch Technologies), we custom designed 20-base oligonucleotide DNA probes that were complementary to the antisense sequence of the *LTR-CMV-EGFP-WPRE-LTR* integrated lentiviral cassette.

Unlike the 3′-RACE, 5′-RACE, and strand-specific RT-PCR experiments that might detect intact or partially degraded antisense transcripts, RNA-FISH primarily detects long transcripts that can accommodate the binding of dozens of fluorescently labeled complementary probes. Alternatively, it is possible that divergent transcription at the *CMV* promoter could generate short antisense ncRNAs (Core et al. 2008; Seila et al. 2008) that are requisite for TGA instead of longer antisense lncRNAs that traverse the entire *CMV-GFP* lentiviral cassette. However, degraded RNA fragments from the antisense lncRNAs or short antisense ncRNAs that arise from within the *CMV* promoter would likely be too short and unstable for detection by RNA-FISH, even if these smaller RNA products were detected by PCR-based techniques.

**FIGURE 6.**
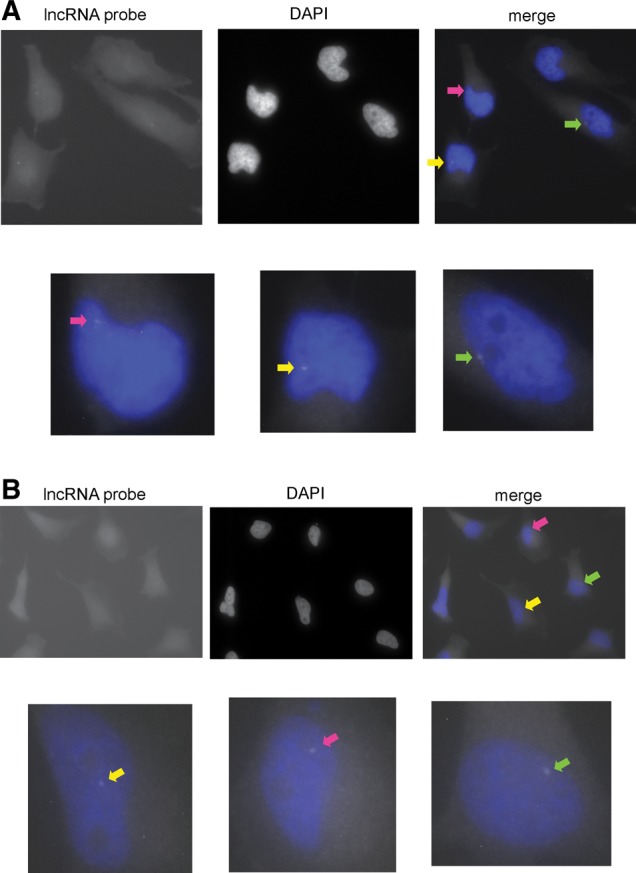
FISH analysis reveals a single antisense lncRNA molecule in the nucleus for clones C5 and E3. (*A*) RNA-FISH to detect antisense lncRNAs that traverse the *CMV-EGFP* reporter cassette for the C5 clone. (*Upper left*) antisense lncRNA; (*upper middle*) DAPI staining; (*upper right*) merged. The *bottom* panel shows the enlarged image of the cells indicated by the color arrows. The probes were custom designed to tile the whole reporter cassette sequence. (*B*) RNA-FISH to detect antisense lncRNAs that traverse the *CMV-EGFP* reporter cassette for the E3 clone. (*Upper left*) antisense lncRNA; (*upper middle*) DAPI staining; (*upper right*) merged. The *bottom* panel shows the enlarged image of the cells indicated by the colored arrows. The probes were custom designed to tile the whole reporter cassette sequence.

A single RNA-FISH spot was detected in approximately half of the cells for clone C5 (Fig. 6A). This spot likely corresponds to the site of active transcription and, due to the single-molecule resolution of RNA-FISH, indicates at least one nascent antisense lncRNA transcript. Moreover, we observed either zero or one single spots per cell in nearly >95% of C5 cells, and >90% of these transcripts were localized in the nucleus. We next applied RNA-FISH on the cells for clone E3. We previously observed that TGA could be triggered in clone E3, but the steady-state levels of antisense RNA were approximately 100-fold lower than C5 (Fig. 4A). Surprisingly, RNA-FISH revealed a comparable level full-length antisense lncRNA for E3 relative to C5 (Fig. 6B). In E3 cells, we observed a single RNA-FISH signal in ∼40% of total cells, and >90% of total cells had either zero or one RNA-FISH signal. Like C5, the detected FISH signals for E3 occurred primarily in the nucleus. Therefore, clones C5 and E3 appeared to have similar levels of lncRNA transcripts by RNA-FISH, with most cells having either one or zero lncRNA-FISH signals. Therefore, we hypothesize that the signals from RNA-FISH represent nascent lncRNAs that are still localized at their site of transcription. Combining the results from strand-specific RNA-FISH (Fig. 6) and RT-PCR (Fig. 4A), we found that two clones C5 and E3 have similar nascent transcription activity of antisense lncRNAs although the steady-state products are at a significantly different level.

### Promoter-associated antisense lncRNAs mediate TGA in *cis*

**FIGURE 7.**
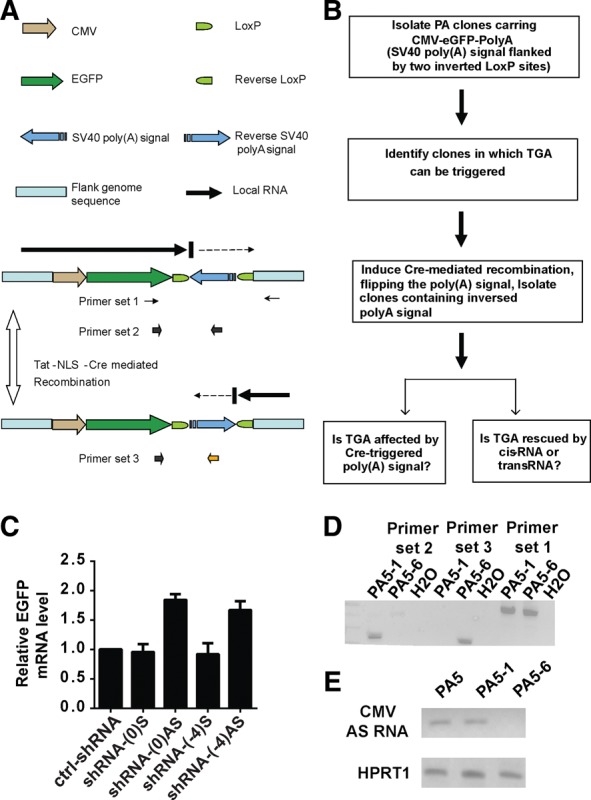
Model to block promoter-associated nascent antisense lncRNA. (*A*) Schematic of the *CMV-EGFP-SV40pA* vector for Cre-induced poly(A) inversion. (*B*) Strategy for generating PA clones and testing the role of nascent antisense RNAs in TGA. (*C*) Quantitative RT-PCR for *eGFP* mRNA 4 d after treatment with irrelevant shRNA (ctrl-shRNA), shRNA(0)S, shRNA(0)AS, shRNA(-4)S, and shRNA(-4)AS in clone PA-5. The shRNA(0)AS and shRNA-(-4)AS are designed to target the promoter-associated antisense lncRNA, while shRNA(0)S and shRNA-(-4)S target in the sense direction. (*D*) PCR genotyping to confirm that the poly(A) signal is inverted in clone PA-5-6, in contrast to clone PA-5-1 and the parent clone PA-5. (*E*) Strand-specific RT-PCR to detect promoter-associated antisense RNAs in clones PA-5, PA5-1, and PA5-6. Clone PA5-6 contains an inverted SV40-poly(A) sequence.

Several lines of evidence have suggested that nascent antisense lncRNAs, rather than the steady-state lncRNA transcripts, were requisite for TGA-inducing shRNAs. First, clones C5 and E3, both with exhibited shRNA-triggered TGA, had similar robust transcription activity (Fig. 6A,B), despite approximately 100-fold difference in steady-state antisense RNA (Fig. 4A). Furthermore, the Ago2 cleavage activity was not required for the induction of TGA and the cleavage of steady-state antisense RNA was not detected after TGA induction (Fig. 4B,C). Therefore, we next asked whether nascent promoter-associated antisense lncRNAs, rather than matured antisense RNAs at steady state, mediated shRNA-triggered TGA. To address this question, we designed a system to unambiguously determine the role of nascent antisense RNAs by blocking antisense transcription through the lentiviral vector. Using a similar *CMV-EGFP* lentivirus as described above, we engineered the 3′ terminal of reporter gene cassette with a SV40-poly(A) signal that was flanked by two inverted LoxP sites (Fig. 7A,B). After Cre-mediated recombination, the SV40-poly(A) signal was designed to flip into the reverse orientation, leading to transcriptional termination of antisense lncRNAs. We isolated HeLa clones transduced with the modified vector (*LTR-CMV-EGFP-WPRE-LoxP-SV40pA-invLoxP-LTR*) and identified one clone (named PA5) that was responsive to shRNA-induced TGA (Fig. 7C). Using 5′-RACE PCR and FISH, we identified an antisense lncRNA originating within the neighboring genomic sequences that was transcribed into the lentiviral integration site in PA5 cells (Supplemental Fig. S6A,B).

After treating PA5 clones with Tat-NLS-Cre protein, we obtained two subclones (PA5-1 and PA5-6). We determined that PA5-1 was the same genotype as the parent clone PA5, whereas PA5-6 had an inverted poly(A) signal (Fig. 7D). The integration sites in PA5-1, PA5-6, and the parent clone PA5 were not disrupted after Cre-induced recombination (Supplemental Fig. S7A). As expected, the poly(A) inversion led to the truncation of the local antisense lncRNAs, which were not detected by strand-specific RT-PCR in PA5-6, but were present in clone PA5-1 and the parent clone PA5 (Fig. 7E). Additionally, the truncation of antisense lncRNAs did not significantly affect the *eGFP* mRNA or protein levels in PA5-6 compared with PA5-1 (Supplemental Fig. S7B,C).

**FIGURE 8.**
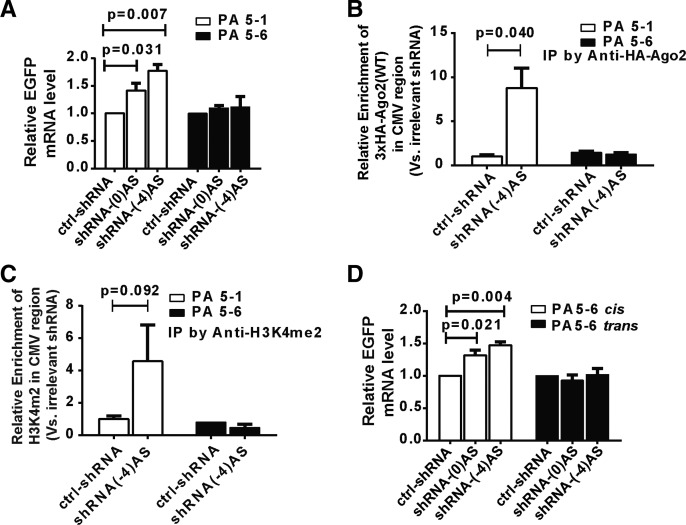
Nascent promoter-associated antisense lncRNAs are required for shRNA-triggered TGA. (*A*) qRT-PCR was used to measure *eGFP* mRNA in clones PA5-1 and PA5-6 4 d after treatment with irrelevant shRNA (ctrl-shRNA) or TGA-inducing shRNAs (shRNA-(0)AS and shRNA-(-4)AS). Both clones are derived from clone PA5 and share the same integration site, but clone PA5-6 has an inverted poly(A) sequence designed of the nascent antisense RNA. Statistical analysis in this study was performed using a two-tailed *t*-test. (*B*) ChIP analysis of Ago2 recruitment to the *CMV* promoter for clones PA5-1 and PA5-6 treated with control (ctrl-shRNA) or TGA-inducing [shRNA-(-4)AS] shRNA. Immunoprecipitated genomic DNA was quantified by qPCR. In this study, all experiments were repeated three times independently and data are represented as mean ± SD. (*C*) ChIP analysis of epigenetic marker H3K4me2 at the *CMV* promoter for clones PA5-1 and PA5-6 treated with control (ctrl-shRNA) or TGA-inducing [shRNA-(-4)AS] shRNA. Immunoprecipitated genomic DNA was quantified by qPCR. In this study, all experiments were repeated three times independently and data are represented as mean ± SD. (*D*) Rescue of TGA phenotype in clone PA5-6. qRT-PCR was used to measure *eGFP* mRNA in clones PA5-6 after rescue using Tat-NLS-Cre recombinant protein (white bars) to restore the *cis*-acting function of the antisense lncRNA. The *trans*-acting function of antisense lncRNA was by ectopic expression of *CMV* antisense lncRNA (black bars).

Strikingly, blocking the nascent antisense lncRNA in clone PA5-6 abolished the shRNA-triggered enhancement of eGFP expression in cells transfected with either shRNA-(0)AS or shRNA-(-4)AS (Fig. 8A). The insensitivity to TGA for PA5-6 was also indicated by no changes in the localization of Ago2 at the *CMV* promoter (Fig. 8B) or in the detection of H3K4 dimethylation in TGA-inducing conditions (Fig. 8C), in contrast to clone PA5-1. This experiment indicates that antisense lncRNA that traverse the entire *CMV-EGFP* region, rather than short antisense ncRNAs from divergent transcription within the *CMV* promoter, are requisite for TGA.

To rescue TGA in clone PA5-6, we first restored the transcription of lncRNAs in the antisense orientation of the *CMV* promoter, which corresponds to reestablishing the lncRNAs in a *cis*-mode. Cre-treatment to clone PA5-6 resulted in ≈70% of total cells inverting the poly(A) back to the original orientation of the parent clone PA5 (Supplemental Fig. S7D). This process did not significantly alter the expression of EGFP mRNA and the steady-state level of antisense *CMV* was partially restored relative to PA5-1 (Supplemental Fig. S7E,F). Importantly, TGA was rescued in Cre-treated PA5-6 cells, consistent with the requirement of an antisense lncRNA for the TGA mechanism (Fig. 8D, left columns). In a different rescue experiment, we over-expressed RNA transcripts that were antisense to the *CMV* promoter into cells by transfection, which provided the antisense lncRNA whose sequence has covered the entire *CMV-EGFP* region in *trans*. This transfection yielded over 150-fold increase in the level of antisense lncRNA compared with the mock transfection (Supplemental Fig. S7G). However, these ectopically expressed antisense lncRNAs failed to rescue shRNA-mediated TGA in PA5-6 cells (Fig. 8D, right columns). Thus, restoration of the transcription of the antisense lncRNA in *cis* rescued a TGA effect, while ectopic expression of lncRNAs in *trans* failed to rescue TGA.

## DISCUSSION

The observation that small RNA can regulate gene expression by inducing the chromatin modulation has been well established in plant and yeast (Grewal 2010; Zhang and Rossi 2011). Similarly, small RNA-induced TGA has been reported in mammalian systems (Chu et al. 2010; Watts et al. 2010; Voutila et al. 2012; Matsui et al. 2013; Reebye et al. 2013a,b), though the molecular mechanisms have not been fully elucidated. In these previous studies, TGA has been induced by two or more shRNAs that target the promoter sequence (Chu et al. 2012). Moreover, mammalian TGA requires promoter-associated lncRNAs, the recruitment of a small RNA–Ago2 complex to the promoter region, and the induction of histone di- or trimethylation of lysine 4 (Li et al. 2006; Janowski et al. 2007; Chu et al. 2010).

In this study, we analyzed small RNA-induced TGA using a *CMV-EGFP* reporter gene system in order to further elucidate the factors and mechanisms of TGA. We tested shRNAs that targeted antisense transcripts at the 5′ region of *CMV* promoter, and we identified two that triggered downstream gene activation (Fig. 2A; Supplemental Fig. S1B). Consistent with other reports of siRNA-triggered TGA (Han et al. 2007; Janowski et al. 2007; Hawkins et al. 2009), the *CMV-EGFP* gene activation is dependent on Ago2 (Fig. 3B,C) and accompanied with histone modification (Fig. 3D). Additionally, we have demonstrated that TGA is also dependent on local, nascent, antisense lncRNA transcripts (Fig. 8A), but is not affected by the ectopic expression of antisense lncRNA (Fig. 8D). Moreover, the requirement of nascent antisense lncRNAs is not sufficient for TGA, as some clones with antisense lncRNAs do not undergo shRNA-induced TGA (Supplemental Figs. S1B, S3B). These findings demarcate a clear boundary between a canonical TGA induced by small RNAs and other indirect off-target effects of PTGS.

The shRNAs in this study were designed so that the “bottom strand” in Figure 1C was preferentially selected as the guide strand (Fig. 1D). Hence, the shRNA-“AS” hairpin would target antisense transcripts while the shRNA-“S” hairpin would have complementarity with sense transcripts within the *CMV* promoter. We observed the strongest induction of EGFP expression in clones C5, D1, and E3 using antisense-targeting shRNAs [shRNA-(0)AS and shRNA-(-4)AS], but the sense-targeting shRNA counterparts [shRNA-(0)S and shRNA-(-4)S] failed to enhance EGFP expression (Fig. 2A). In fact, for clone D1, shRNA-(-4)S slightly reduced EGFP expression. Similar trends were observed between antisense-targeting shRNA-(0)AS and sense-targeting shRNA-(0)S for clones C6, C8, D2, D3, and E4, where the “S” hairpin exhibited a slight inhibitory effect on EGFP expression (Supplemental Fig. S1B). It is established that small RNAs directed against a sense promoter-associated transcript can induce transcriptional gene silencing (TGS) on that promoter (Han et al. 2007; Hawkins et al. 2009; Tan et al. 2009; Yue et al. 2010), and like TGA, this process involves an Argonaute protein that is loaded with the small RNA and associated with chromatin-modifying factors (Kim et al. 2006; Weinberg et al. 2006; Chu et al. 2010). Therefore, the shRNA-“AS” configuration may induce TGA within certain chromatin environments with antisense ncRNA transcripts, while the shRNA-“S” configuration may induce TGS in other chromatin environments having sense promoter-associated transcripts. This is consistent with an observation in which one shRNA against the *VEGF-A* promoter induced epigenetic silencing, while another shRNA against the *VEGF-A* promoter enhanced chromatin activation (Turunen et al. 2009). We have also observed that a single-nucleotide shift may abrogate a TGA response, and may even lead to a silencing effect (Supplemental Fig. S1A,B). However, we note similar observations from Janowski et al. (2007), in which a one base-shift in the duplex RNA sequence seemed to have a similar effect on inducing TGA or even TGS.

In contrast to previous TGA models that have been based on endogenous gene loci, we have adopted a model system that uses a single *CMV* promoter that is located across different integration positions and chromatin environments. Using this model, we have found that antisense RNA transcription is one of the indispensible constituents of the required chromatin condition (Figs. 6A,B, 8A). This requirement was established by strand-specific RT-PCR, RNA-FISH, and RACE PCR for the five clones in this study (C5, D1, E3, PA5, and PA5-1) that exhibited shRNA-induced TGA. Nevertheless, this observation was not entirely unexpected, as the majority of human genome has the potential to transcribe RNA (Cheng et al. 2005; Katayama et al. 2005). The antisense transcripts required for TGA could represent lncRNAs that originated from within the genomic region and were transcribed through the lentiviral gene cassette or shorter antisense ncRNAs that originated from within the *CMV* promoter region. The second type of antisense ncRNAs have been identified in many endogenous promoter regions at the genome-wide level (Core et al. 2008; Seila et al. 2008). In this study, we have no direct evidence that indicates a requirement for short AS-RNAs for TGA, but we do demonstrate that lncRNAs are required. Specifically, in clone PA5-6, which lost its ability for TGA induction after the inversion of the SV40-poly(A) sequence (Fig. 8A), transcription of antisense lncRNAs was blocked (Fig. 8B), but transcription of short AS-RNAs would not have been affected. Nevertheless, it should be noted that the short AS-RNA may contribute to detection of antisense transcripts by strand-specific RT-PCR, but do not contribute to RNA-FISH signal due to their short length (Figs. 4A, 6; Supplemental Fig. S3A,B). The existence of short AS-RNA can explain the observation that E3 has significantly lower steady-state antisense lncRNA levels (Fig. 4A), whereas it has similar antisense transcription activity as C5 as measured by RNA-FISH (Fig. 6A,B).

Previous studies have shown that antisense lncRNAs can repress gene expression in the sense direction through the recruitment of histone-modifying enzymes (Khalil et al. 2009; Gupta et al. 2010; Tsai et al. 2010; Kretz et al. 2013; Maamar et al. 2013). Our system indicates a similar role of antisense lncRNAs for TGA, and is distinct from other types of antisense regulation, such as transcriptional interference or translational inhibition. However, in this model for TGA, we have demonstrated that the repressive role of the antisense lncRNA can be reversed by the introduction of a complementary small RNA. This mechanism is not related to the Ago2-mediated cleavage of the antisense lncRNA, since its levels were not affected when TGA was triggered (Fig. 4B). Likewise, RNA triggers with mutations that compromise Ago2-mediated cleavage can still induce TGA (Fig. 4C), consistent with the notion that the Ago2–shRNA complex does not destroy the lncRNA. Finally, Cre-induced inversion of the poly(A) sequence prevents expression of antisense lncRNAs, and no significant changes were observed in EFGP expression or in chromatin status between clones PA5-1 and PA5-6 (Supplemental Figs. S7B,C, 8C), which suggests a mechanism distinct from transcriptional interference or translational inhibition. Taken together, we concluded that TGA is not a derepression of the antisense lncRNAs by the Ago2–siRNA complex and that elimination of the antisense lncRNA is insufficient for gene activation. In contrast, our model supports a functional role of antisense lncRNAs for modulating the neighboring sense gene expression, in which the lncRNA serves as a scaffold to recruit the Ago2–shRNA complex and other chromatin remodeling factors. In this regard, the role of lncRNA in mammalian TGA is a reminiscent of yeast and plant TGS (Buhler et al. 2006; Wierzbicki et al. 2008).

One of the primary objectives in our TGA system was to determine how the antisense lncRNA interacted with the target genomic loci (i.e., in *cis* or in *trans*), which is a critical factor in understanding a TGA mechanism for other genes. Our findings strongly support a *cis*-lncRNA model for small RNA-induced TGA, in which the antisense lncRNA functions as a molecular scaffold at the site of transcription. First, we have detected antisense lncRNAs for all clones that exhibit TGA, yet we have not observed a correlation between the steady-state lncRNAs and the induction of TGA (Supplemental Figs. S3B, 4A, 6A,B). Second, we have found that terminating the transcription of antisense lncRNAs can abrogate the induction of TGA, and TGA can only be rescued by providing antisense lncRNAs in *cis*-mode, not in *trans*-mode (Fig. 8A–C). These two findings suggest that nascent lncRNA transcripts, rather than the steady-state levels of the antisense lncRNAs, are involved in the TGA mechanism. As indicated by the *cis*-model in our study, the existence or absence of the antisense lncRNAs will determine whether TGA can be triggered (Fig. 8A), whether an active chromatin signature can be enriched (Fig. 8B), and whether Ago2-shRNA can be recruited to the promoter region (Fig. 8C). Taken together, the lncRNAs in this system mediate TGA in *cis* and not in *trans*, serving as a molecular scaffold for an Ago2–shRNA complex and chromatin-modifying enzymes. This model is consistent with a recent report of miRNA-triggered TGA at the *COX-2* promoter (Matsui et al. 2013).

While our model for TGA indicates the requirement of a *cis*-acting lncRNA, this requirement may not apply for all small RNA-triggered TGA. Numerous examples of *trans*-acting lncRNAs have been documented, though most of these have not yet been examined as targets for small RNA-triggered TGA (for review, see Guttman and Rinn 2012). In some cases, *trans*-acting lncRNAs that silence genes epigenetically might become derepressed by complementary small RNAs inside the nucleus. Recently, the Morris lab reported gene activation of the HIV promoter using small RNAs that targeted a nuclear, viral-encoded antisense lncRNA, which effectively suppressed the antisense lncRNA and reversed epigenetic silencing (Saayman et al. 2014). Nevertheless, compared with models for TGA that involve *trans*-lncRNA, a *cis*-lncRNA TGA model requires only nascent transcription and is not constrained by evolutionary conservation for the lncRNAs (such as antisense ncRNAs from pseudogenes) and the targeted gene or promoter sequences. This is consistent with our finding that TGA can be induced on the *CMV* promoter, which is not homologous to any human genomic sequences. The intrinsic nature of a *cis*-regulatory mechanism may offer a method for exogenously activating a particular gene using sequence-specific small RNAs, which implicates the powerful, gene-specific potential of small RNA-induced TGA.

## MATERIALS AND METHODS

### Cell culture and cell cloning

HeLa cells (ATCC) were grown in DMEM (Irvine Scientific) supplemented with 10% FCS (Irvine Scientific) and 1 mM l-glutamine. For cell cloning, about 800–1000 cells were trypsinized and disrupted into single cells which were seeded into one 10-cm tissue culture dish (BD Falcon) and cultured for 10 d until the clones are visible. The cell clones were marked under phase-contrast microscope and picked up by a cotton swab. After isolation, the clonal cells were cultured under the tissue culture conditions described above. For selection of neomycin-resistant cells, the culture medium is supplemented with 500 µg/mL G418 (Sigma) for at least 1 wk.

### Statistical analysis

Statistical analysis used a two-tailed *t*-test. Statistical *P*-values are indicated in the figures. Error bars represent standard deviations of three independent experimental measurements.

### Virus packaging and lentiviral infections

HEK 293T cells were used to package lentivirus by cotransfecting the lentiviral vector with packaging plasmids pCHGP-2 (HIV Gag/Pol), pCMV-Rev (HIV Rev), and pCMV-G (VSV-G). Lentivirus packaging and virus titering assays were performed as previously described (Aagaard et al. 2007). Lentivirus was concentrated by ultracentrifugation (24,500 rpm at 4°C for 2 h) and infected on HeLa cells at an approximate MOI of 0.2.

### Plasmids and vector construction

The plasmids expressing U6-driven shRNAs are previously described (Aagaard et al. 2007). The plasmids expressing 3xHA tagged Ago2 and Ago2 Q633R/H634P mutant were constructed by PCR-amplification of the gene and cloning it downstream from the human ubiquitin C promoter. The CMV antisense RNA overexpression vector (pCMV-RFP-TRE) was constructed by cloning a TRE-miniCMV promoter (PCR cloned from pTRE-Tight vector, Clontech) downstream from the CMV-RFP gene in a pHIV-7 lentiviral plasmid. All sequences and plasmids are available upon request.

### Transfection and Tat-NLS-Cre treatment

For transfection of plasmids that express U6-shRNAs, approximately 180,000 HeLa cells were plated per well in 12-well plates (Costar) 16–24 h before transfection. For the transfection in ChIP experiments, approximately 3 million cells were plated in 10-cm dish (BD Falcon) 24 h before transfection. For each well in 12-well plate, 2 µL Lipofectamine 2000 (Invitrogen) was mixed with 0.75 µg plasmid DNA in 300 µL Opti-MEM (Invitrogen). For each 10 cm dish, 15 µg DNA was mixed with 40 µL Lipofectamine in 4 mL Opti-MEM. The Lipofectamine–DNA mixture was incubated with cells for 6 h until the medium was replaced with normal culture medium described above. Twenty-four hours later, one-third of the transfected cells per 12-well were trypsinized and transferred into one 6-well. Total RNA was extracted 4 d after transfection. For Tat-NLS-Cre (Excellgen) treatment, Cre protein was added into 1 mL medium at a final concentration of 1 µM. The medium was applied on 200,000 cells seeded in one 12-well. The medium was changed 24 h later after incubation.

### Quantitative RT-PCR

Total RNAs were extracted by using RNA STAT-60 (IsoTex Diagnostics) according to the manufacturer's instructions. About 1–2 µg total RNA was reverse-transcribed into cDNA (complementary DNA) by using random hexamer primers (Invitrogen) and Moloney murine leukemia virus reverse transcriptase (MMLV-RT, Invitrogen). For strand-specific reverse transcription, 2 pmol strand-specific primer is used to generate cDNA. As a negative control, each prepared RNA sample was processed without MMLV-RT. About 1/10 of total cDNA was subjected to qRT-PCR analysis by mixing with 2× IQ-SYBR Green Supermix (Bio-Rad, C1000 Thermal Cycler, Bio-Rad). *EGFP* and antisense RNA expression levels were normalized by the housekeeping gene *HPRT1*. PCR conditions were 95°C for 5 min, followed by 40 cycles of 95°C for 30 sec, 60°C for 30 sec, and 72°C for 30 sec (Supplemental Table S3 for primer sequences).

### Chromatin immunoprecipitation (ChIP)

ChIP was performed using the EZ-ChIP kit (Millipore, #17-371) per the manufacturer's instructions with minor modification. Approximately 20 million cells were treated with hypotonic buffer and dounced on ice to extract nuclei. Nuclei were resuspended in 1 mL lysis buffer for sonication. Chromatin was reverse cross-linked, purified, and quantified by spectrophotometry (NanoDrop 2000). Approximately 20 µg chromatin was used for Ago2 immunoprecipitation. For histone H3 trimethyl K9 and histone H3 dimethyl K4, 5 µg chromatin was used. The reverse-crosslink was performed at 65°C overnight and the precipitated DNA was quantified by qPCR. Antibodies used for ChIP included anti-HA (Abcam, ab9110) and anti-Histone H3 (dimethyl K4) (Abcam, ab7766).

### Inverse PCR

The inverse PCR was performed according to the previous report (Triglia 2000) with minor modification. Approximately 1 µg genomic DNA was digested by either EcoRI or XbaI restriction enzymes (NEB) at the final concentration of 0.2 units/µL in the total volume of 50 µL. DNA was purified by phenol–chloroform extraction and ethanol precipitation. DNA 2 ng/µL was ligated at 16°C overnight by T4 DNA ligase (NEB) at the final concentration of 16 units/µL in a total volume of 100 µL. Ligation products were heated at 95°C water bath for 5 min. Approximately 5 µL of the ligation products were amplified by PCR using JumpStart REDAccuTaq LA DNA Polymerase (Sigma). The PCR condition is 96°C for 60 sec, followed by 30 cycles of 94°C for 15 sec, 60°C for 30 sec, and 68°C for 5 min. The final extension is 20 min.

### Northern and Southern blot analyses

Total RNA was isolated using RNA STAT-60 according to the manufacturer's instructions. About 10 µg total RNA was fractionated in denatured 8% PAGE and transferred onto a Hybond-N+ membrane (Amersham Pharmacia Biotech). The probes are synthesized 25-nt DNA oligos that are ^32^P-radiolabeled and complementary to target sequences. Hybridization was performed for 16 h at 37°C. Southern blots were performed according to standard protocols (Green et al. 2012) and 20 µg of genomic DNA was digested by XbaI (NEB) and loaded into each lane.

### Western blot analysis

The total protein was extracted from the cell by incubating in the lysis buffer supplemented with 1× cocktail of protease inhibitors (Roche). The protein concentration was quantified by the Bradford method (Bio-Rad, Protein assay dye). Approximately 10–20 µg of total protein extract was analyzed by electrophoresis using a 7% SDS-PAGE gel at 100 V for 2 h, and electro-blotted to a Hybond-P PVDF transfer membrane (GE Healthcare) for 90 min at 80 V. For blotting with the Ago2 antibody, the membrane was blocked with 5% BSA with 0.5% Tween-20 at room temperature for 1 h. Alternatively, membrane was incubated in the blocking buffer (10 mM Tris–HCl, pH 8.0, 150 mM NaCl, 0.05% Tween-20, 5% powdered milk solution) for 1 h. Primary antibodies were diluted in the blocking buffer and added to the membrane overnight at 4°C with gentle rotating. Horseradish Peroxidase-Conjugated Secondary Antibody (Santa Cruz Biotechnology) diluted in the blocking buffer was added to the blots the next day, and the membranes were incubated for 1 h with shaking. ECL Plus Western Blotting Detection Reagents (RPN2132; GE Healthcare) were used as described by the manufacturer's protocol, and the resulting signal was detected by autoradiography. Primary antibodies included anti-AgoI (07-599; Millipore), anti-AgoI (04-083; Millipore), anti-HA (Abcam, ab9110), anti-Ago2 (Cell signal, #2897). The conjugated secondary antibodies included anti-mouse IgG-peroxidase (A3682; Sigma), anti-rabbit IgG-peroxidase (A0545; Sigma), and anti-goat IgG-peroxidase (A-5420; Sigma).

### 5′ RACE PCR

Approximately 5 μg of total RNA was sequentially treated with 1 unit/μL calf intestine phosphatase (CIP, NEB) and 1 unit/μL Tobacco Acid Pyrophosphatase (TAP, Epicentre) for 1 h at 37°C. During mock treatment, the sample was incubated with buffer without enzyme. The RNA was purified by phenol-extraction and recovered by ethanol precipitation before the following treatment. The RNAs were then ligated with adaptor RNAs using RNA Ligase-1 (NEB). The ligation products were subjected to reverse transcription and nested PCR. The PCR products were analyzed by 1% agarose gel and sequenced by TA cloning or sequenced directly.

### 3′ RACE PCR

About 3 μg total RNAs were treated by 1 μL *Escherichia coli.-*Poly(A) polymerase (10 unit/μL NEB) at 37°C for 30 min. The poly(A)-tailed RNAs were reverse transcribed and amplified by nested PCR. The PCR products were analyzed by 1% gel and sequenced by TA cloning.

### RNA-FISH

The FISH was performed according to the Stellaris manufacture's protocol and previous reports (www.biosearchtech.com/stellarisprotocols). The probes were labeled with Quasar 670. After hybridization of probes, the cells were counter-stained by DAPI and mounted with Vectashield Mounting Medium (Vector Labs, catalog #H1000) for imaging.

## SUPPLEMENTAL MATERIAL

Supplemental material is available for this article.

## Supplementary Material

Supplemental Material
