# Supplementary material for: The role of antisense long noncoding RNA in small RNA-triggered gene activation
Source: RNA. 2014 Dec;20(12):1916–28. doi: 10.1261/rna.043968.113 (PMC4238356; doi:10.1261/rna.043968.113)
Supplement: Supplemental Material [file supp_20_12_1916__index.html]

The role of antisense long noncoding RNA in small RNA-triggered gene activation — The role of antisense long noncoding RNA in small RNA-triggered gene activation — Supplemental Material 

# The role of antisense long noncoding RNA in small RNA-triggered gene activation

## Supplemental Material

**Files in this Data Supplement:**

- Supp Material.pdf
