# Supplementary material for: The role of antisense long noncoding RNA in small RNA-triggered gene activation
Source: RNA. 2014 Dec;20(12):1916–28. doi: 10.1261/rna.043968.113 (PMC4238356; doi:10.1261/rna.043968.113)
Supplement: Supplemental Material [file supp_043968.113_SuppMaterial.pdf]

Supporting information for:

The role of antisense long non-coding RNA in small RNA-triggered gene activation

Xizhe Zhang[1], Haitang Li[2], John C. Burnett[1]*, and John J. Rossi[1,2]*

* correspondence should be addressed to J.C.B. (jburnett@coh.org) or J.J.R. (jrossi@coh.org)

Contents

Supplemental Figures S1-S7

Fig. S1. The effects of promoter-targeted shRNAs in eight *CMV-eGFP* HeLa clones.
Fig. S2. Involvement of Ago2 in shRNA-induced TGA.
Fig. S3. Promoter-associated antisense RNAs are detected in *CMV-eGFP* clones.
Fig. S4. Mapping of antisense transcription in D1 clone by RACE PCR.
Fig. S5. Mapping of antisense transcription in E3 clone by RACE PCR.
Fig. S6. Detection of antisense lncRNAs in PA5 cells.
Fig. S7. Antisense lncRNAs mediate TGA in *cis*.

Supplemental Tables S1-S3

Table S1. The integration sites of CMV-eGFP clones.
Table S2. The sequences of shRNAs used in this study.
Table S3. Primers used for qRT-PCR, ChIP, and genotyping.

Supporting Text S1-S5

Text S1. Sequencing result for C5-AS1 lncRNAs.
Text S2. Sequencing result for the antisense RNAs in C5 cells detected by 3' RACE PCR.
Text S3. Sequencing result for 5' RACE PCR result of D1 antisense lncRNAs.
Text S4. Sequencing result for the antisense RNAs in E3 cells detected by 3' RACE PCR.
Text S5. Sequencing result for the antisense RNAs in PA5 cells detected by 5' RACE.

Captions for Supplementary Figures S1-S7

Figure S1. The effects of promoter-targeted shRNAs in eight *CMV-eGFP* HeLa clones.

S1A. The effect of *CMV*-targeting shRNAs on *eGFP* expression in HeLa clone D1. Cells were treated with shRNAs that targeted the *CMV* promoter region in the sense (S) and antisense (AS) orientations, as measured by qRT-PCR. Each shRNA targets a 19-nucleotide sequence, and the target sequence is shifted by a single nucleotide for each shRNA. For instance, the target sequence for shRNA-(-1)AS lies one nucleotide upstream of shRNA-(0), while the target sequence for shRNA-(1)AS is positioned one nucleotide downstream of shRNA-(0). Sequences of the shRNAs are provided in Supplementary Table 2.

S1B. *EGFP* mRNA levels for seven *CMV-eGFP* clones after transfection with promoter-targeting shRNAs. *CMV-eGFP* HeLa clones C3, C4, C6, C8, D2, D3, and E4 were analyzed for enhanced EGFP expression by qRT-PCR after treatment with shRNA-(-4)AS, shRNA-(-4)S, shRNA-(0)AS, and shRNA-(0)S, as performed in Figures 2A and S1A.

S1C. Western blot of EGFP protein levels for control-shRNA (left column), shRNA-(0)AS (center column), and shRNA-(-4)AS (right column) for HeLa clone C5. α-tubulin is the loading control.

S1D. Constitutive *eGFP* mRNA levels for seven *CMV-eGFP* clones. EGFP expression was measured by qRT-PCR in cells without shRNA transfection for each of the seven *CMV-eGFP* HeLa clones from S1B.

Figure S2. Involvement of Ago2 in shRNA-induced TGA.

S2A. Quantitative RT-PCR to detect TGA response in normal D1 clones (black bars) or D1 clones with depleted levels of endogenous Ago2 (gray bars). Anti-Ago2 siRNAs and plasmids expressing TGA-inducing shRNAs were co-transfected into cells. The RNA samples were extracted four days post-transfection for analysis.

S2B. Western blot of 3XHA-Ago2 protein in HeLa C5 cells using an antibody against the HA tag. From right to left, cells were transfected with control shRNA and 3XHA-Ago2 plasmid (left column), shRNA-(0)AS and 3XHA-Ago2 plasmid, shRNA-(-4)AS and 3XHA-Ago2 plasmid, or a 3XHA-control vector.

S2C. Western blot of endogenous and overexpressed Ago2. Wild type 3XHA-Ago2 (center column) and 3XHA-Ago2 Q633R/H634P mutant (right column) proteins are expressed in C5 cells at a similar level to endogenous Ago2 (left column) as detected by Western blot using antibody against the endogenous Ago2.

S2D. Wild type Ago2 and mutant Ago2 incorporate TGA-triggering shRNA. Wild type 3XHA-Ago2 (left column) or 3XHA-Ago2 Q633R/H634P mutant (center column) proteins were immunoprecipitated, followed by Northern blot of the shRNA using 25-nt 32P-radiolabeled probes. The size of the band (≈20 bp) suggests that the shRNA has been processed into a single-stranded RNA that was incorporated into Ago2 or the Q633R/H634P Ago2 mutant.

S2E. Ago2 chromatin immunoprecipitation at the *GAPDH* promoter. ChIP for 3XHA-Ago2 (center column) or 3XHA-Ago2 Q633R/H634P mutant was followed by qPCR against the *GAPDH* promoter region in C5 clones transfected with shRNA-(-4)AS.

S2F. Wild type and Q633R mutant Ago2 are enriched at the *CMV* promoter. ChIP for

3XHA-Ago2 (center column) or 3XHA-Ago2 Q633R/H634P mutant was followed by qPCR against the *CMV* promoter region in C5 clones transfected with shRNA-(-4)AS.

Figure S3. Promoter-associated antisense RNAs are detected in *CMV-eGFP* clones.

S3A. Promoter-associated antisense RNAs are detected in C5, D1, and E3 by strand-specific RT-PCR. As a negative control, each prepared RNA sample was processed without MMLV-RT, which yielded no detectable PCR product for each clone. Primer sequences are described in Supplementary Table 3.

S3B. Promoter-associated antisense RNA levels are represented as RNA copies per cell in clones C5, D1 and E3, as detected by strand-specific qRT-PCR (See methods for details). As a negative control, each prepared RNA sample was processed without MMLV-RT, which yielded no quantifiable PCR product for each clone. Primer sequences are described in Supplementary Table 3.

S3C. Mutation of shRNA-triggers affects TGA in D1 cells. Levels of *EGFP* mRNA were quantified by qRT-PCR for clone D1 treated with control shRNA, shRNA-(0)AS-M5' (mismatches at nucleotides 1-5) , shRNA-(0)AS-(9-11) (mismatches at nucleotides 9-11), or shRNA-(0)AS-M3' (mismatches at nucleotides 15-19). Primer sequences are described in Supplementary Table 2.

Figure S4. Mapping of antisense transcription in D1 clone by RACE PCR.

S4A. An antisense lncRNA with a mono-phosphate group at the 5' termini is detected in D1 cells by 5' RACE PCR. D1-AS1 originates from the adjacent genomic region 0.75 kb downstream of the 3' terminus of the integrated cassette. CIP/TAP/Ligation (left lane): total RNAs were treated by calf-intestinal alkaline phosphatase (CIP), tobacco acid pyrophosphatase (TAP), and ligated to RNA adaptors before analysis by nested PCR. Only lncRNAs with an intact 5' cap structure can be detected by this method. Ligation (middle lane): total RNAs were mock treated by CIP and TAP sequentially before they were ligated to RNA adaptor and subjected to nested PCR analysis. The lncRNAs with mono-phosphate can be detected by this method. CIP/Ligation (right lane): total RNAs were treated by CIP, ligated to RNA adaptors, and analyzed by nested PCR. This lane shows the general background of 5' RACE. The bands on the gel indicate lengths of nested PCR products, not the absolute lengths of antisense RNA.

S4B. Schematic diagram shows the origination of antisense lncRNAs in D1 cells. The integration site is described in Supplementary Table 1.

Figure S5. Mapping of antisense transcription in E3 clone by RACE PCR.

S5A. Strand-specific RT-PCR detects the antisense lncRNAs that are transcribed into the integration site in E3 cells.

S5B. 3' RACE PCR determines the multiple termini of antisense lncRNAs that are transcribed into the integration site in E3.

S5C. Schematic diagram shows the transcribed-through antisense lncRNAs in E3 cells.

Figure S6. Detection of antisense lncRNAs in PA5 cells.

S6A. 5' RACE PCR detects the antisense lncRNAs that are transcribed into the integration

site in PA5 cells.
S6B. FISH analysis detects single antisense lncRNAs.

Figure S7. Antisense lncRNAs mediate TGA in *cis*.
S7A. PCR genotyping of clones PA5, PA5-1 and PA5-6 using primers that flank the integration junction in chromosome. Results show that all three clones are integrated in the same chromosome sites.
S7B. The *EGFP* mRNA level detected by qRT-PCR in clones PA5-1 and PA5-6.
S7C. Flow cytometry analysis for eGFP expression in HeLa PA5-1 clone (red outline), HeLa PA5-6 clone (blue outline), and control HeLa cells (gray fill).
S7D. Cre-treated PA5-6 were tested for the percentage of cells in which recombination occurs. After treated with Tat-NLS-Cre, recombination occurred in a fraction of the PA5-6 cells. PCR products were subjected to TA cloning and sequencing. About 70% of total cells underwent Cre-mediated recombination, to become the same genotype as the PA5 parent cells.
S7E. There is no change in the eGFP mRNA level before and after treating PA5-6 cells with Cre-recombinase.
S7F. *CMV* antisense lncRNAs were partially recovered after PA5-6 cells were treated with Cre-recombinase.
S7G. A system to provide antisense RNAs in trans: PA5-6 and PA5-1 was first transduced with the retrovirus carrying reverse Tet repressor protein gene (Clontech Tet-On system). At the presence of doxycycline (Dox 1 μg/ml), antisense RNA is expressed driven by downstream *TRE –miniCMV* promoter.

Supplemental Table S1. The integration sites of *CMV-eGFP* clones.

**Clone name**	**Integration sites**	**Relationship to the host gene**	**Host Gene**
C3	Intergenic	N/A	N/A
C4	First Intron	Same Direction	CHPt1
C5	Forth Intron	Opposite Direction	DDI2
C6	Second Intron	Opposite Direction	PLEC1
C8	First Intron	Same Direction	EIF2C3
D1	First Intron	Same Direction	CtGBP1
D2	Intergenic	N/A	N/A
D3	Fourth Intron	Opposite Direction	HtR2C
E3	Intergenic	N/A	N/A
E4	Third Intron	Opposite Direction	VPS13A

Supplemental Table S2. The sequences of shRNAs used in this study.

	**shRNA Name**	**shRNA Sequence** (capital letters represent the shRNA loop)
1	shRNA-(0)S	5' ggaguuccgcguuacauaaCCUACCCAuuauguaacgcggaacucc
2	shRNA-(0)AS	5' uuauguaacgcggaacuccCCUACCCAggaguuccgcguuacauaa
3	shRNA-(-1)S	5' uggaguuccgcguuacauaCCUACCCAuauguaacgcggaacucca
4	shRNA-(-1)AS	5' uauguaacgcggaacuccaCCUACCCAuggaguuccgcguuacaua
5	shRNA-(-2)S	5' auggaguuccgcguuacauCCUACCCAauguaacgcggaacuccau
6	shRNA-(-2)AS	5' auguaacgcggaacuccauCCUACCCAauggaguuccgcguuacau
7	shRNA-(-3)S	5' uauggaguuccgcguuacaCCUACCCAuguaacgcggaacuccaua
8	shRNA-(-3)AS	5' uguaacgcggaacuccauaCCUACCCAuauggaguuccgcguuaca
9	shRNA-(-4)S	5' auauggaguuccgcguuacCCUACCCAguaacgcggaacuccauau
10	shRNA-(-4)AS	5' guaacgcggaacuccauauCCUACCCAauauggaguuccgcguuac
11	shRNA-(1)S	5' gaguuccgcguuacauaacCCUACCCAguuauguaacgcggaacuc
12	shRNA-(1)AS	5' guuauguaacgcggaacucCCUACCCAgaguuccgcguuacauaac
13	shRNA-(2)S	5' aguuccgcguuacauaacuCCUACCCAaguuauguaacgcggaacu
14	shRNA-(2)AS	5' aguuauguaacgcggaacuCCUACCCAaguuccgcguuacauaacu
15	shRNA-(3)S	5' guuccgcguuacauaacuuCCUACCCAaaguuauguaacgcggaac
16	shRNA-(3)AS	5' aaguuauguaacgcggaacCCUACCCAguuccgcguuacauaacuu
17	shRNA-(0)AS-M3'	5' aauacuaacgcggaacuccCCUACCCAggaguuccgcguuaguauu
18	shRNA-(0)AS-M5'	5' uuauguaacgcggauaaggCCUACCCAccuuauccgcguuacauaa
19	shRNA-(0)AS-M(9-11)	5' uuauguaauuuggaacuccCCUACCCAggaguuccaaauuacauaa
20	shRNA-U6	5’ cggagacagcgagcgagagcUUUGUGUAgcucucgcucgcugucuccg

Supplemental Table S3. Primers used for qRT-PCR, ChIP, and genotyping.

**ChIP**	**Primer Sequence**
CMV-F	CAAGTACGCCCCCTATTGAC
CMV-R	AAGTCCCGTTGATTTTGGTG
**Strand Specific RT-PCR**	**Primer Sequence**
F14 (for RT)	CATGACCTTATGGGACTTTC
CMVantisense-F	CACCAAAATCAACGGGACTT
CMVantisense-R	GCTGAACTTGTGGCCGTTTA
**qRT-PCR**	**Primer Sequence**
EGFP-F	ACGTAAACGGCCACAAGTTC
EGFP-R	AAGTCGTGCTGCTTCATGTG
HRPT1-F	TGACACTGGCAAAACAATGCA
HRPT1-R	GGTCCTTTTCACCAGCAAGCT
**Genotyping**	**Primer Sequence**
Primer set1-F	ACATGGTCCTGCTGGAGTTC
Primer set1-R	GGCATTAAAGCAGCGTATCC
Primer set2-F	CGACAACCACTACCTGAGCA
Primer set2-R	GGGAGGTGTGGGAGGTTTT
Primer set3-F	CGACAACCACTACCTGAGCA
Primer set3-R	GCAATAGCATCACAAATTTCACA

Captions for Supporting Text S1-S5

Text S1. Sequencing result for C5-AS1 lncRNAs.
Text S2. Sequencing result for the antisense RNAs in C5 cells detected by 3' RACE PCR.
Text S3. Sequencing result for 5' RACE PCR result of D1 antisense lncRNAs.
Text S4. Sequencing result for the antisense RNAs in E3 cells detected by 3' RACE PCR.
Text S5. Sequencing result for the antisense RNAs in PA5 cells detected by 5' RACE.

Supporting text S1: Sequencing result for C5-AS1 lncRNAs

The sequence underlined is the 5' adaptor.
The sequence in red is the vector sequence.
The starting site for C5-AS1 is represented by the lowercase "t", which is located at the neighboring genomic region about 700 bp from the 3' terminal of the integrated vector.

<u>CGACTGGAGCACGAGGACACTGACATGGACTGAAGGAGTAGAAA</u>tTTTTTGGTAATTACCTT
ATTTTAGTAAAAGTTTGGAAGATTAAAACAGATACACATATGTTTCCAGGCTTAGTGAAGGG
GATGTACCAACTTCAAAGAATTAAAAAGAAATGGCTTTTTACCTAAATAACCTTTTGTTGCT
GGTAGTATAGCTTGTGCTAATTTTTGTAATGTTTAAGTAATGAAATAATTCTACATATCAGG
TCCTTTTTATTTAGAACATCGACTTCCTAATATCATCCGAATAAATACTCTGGACATAGTAG
GTGAAAGTATTTTGTTAACAACTTCTTTCTGGCCAAGCATGGTGGCTCATGCCTGTGGTCCC
AACACTTTGGGAGGCTGAGGTGGGGAGATCACTTGAACCTATGGATTTGAGACCAGCCTGGG
CAGCATGGCAAAACACTTGTTTCTAAAAAAAAAAACCACGGCTGGGCGCAGTGACTCACGCC
TGTAATCCCAACACTTTGGGAGGCTGAGGAAGGGTGGATCACCTGAGGTCAGAAATTTGAGA
CCAGCCTGGCCAACATGGTGAAACCTCATCTCTACTAAAAATACAAAAATTAGCCAGGCATG
GTTGTGGGCGCCTGTAATCCCAGCTACTCGGGAGGCTGAGGCAGGAGAATTGCTTGAAGCCG
GGAAGCAGAGGTTGCAGTGAGCCAAGATTGCACCACTGCACTCCAGCCTGGGCAACAGAGTG
TGCTAGAGATTTTCCACACTGACTAAAAGGGTCTGAGGGATCTCTAGTTACCAGAGTCACAC
AACAGACGGGCACACACTACTTGAAGCACTCAAGGCAAGCTTTATTGAGGCTTAAGCAGTGG
GTTCCCTAGTTAGCCAGAGAGCTCCCAGGCTCA

Supporting text S2: Sequencing result for the antisense RNAs in C5 cells detected by 3' RACE PCR.

The sequence of the 3' terminus of virus vector was shown. The red underlined letters are the detected termini of antisense RNAs that are transcribed into the integration sites. The underlined sequences are the primer binding sites.

CAAAATTTTCGGGTTTATTACAGGGACAGCAGAGATCCAGTTTGGGGATCCTGAGCGGCCGC
AAGGTGACCCGTCTAGAGATGGATCCCCCGGGCTGCAGGAATTCGATGATCCAGTATTACCG
CCATGCATTAGTTATTAATAGTAATCAATTACGGGGTCATTAGTTCATAGCCCATATATGGA
GTTCCGCGTTACATAACTTACGGTAAATGGCCCGCCTGGCTGACCGCCCAACGACCCCCGCC
CATTGACGTCAATAATGACGTATGTTCCCATAGTAACGCCAATAGGGACTTTCCATTGACGT
CAATGGGTGGAGTATTTACGGTAAACTGCCCACTTGGCAGTACATCAAGTGTATCATATGCC
AAGTACGCCCCCTATTGACGTCAATGACGGTAAATGGCCCGCCTGGCATTATGCCCAGTACA
TGACCTTATGGGACTTTCCTACTTGGCAGTACATCTACGTATTAGTCATCGCTATTACCATG
GTGATGCGGTTTTGGCAGTACATCAATGGGCGTGGATAGCGGTTTGACTCACGGGGATTTCC
AAGTCTCCACCCCATTGACGTCAATGGGAGTTTGTTTTGGCACCAAAATCAACGGGACTTTC
CAAAATGTCGTAACAACTCCGCCCCATTGACGCAAATGGGCGGTAGGCGTGTACGGTGGGAG
GTCTATATAAGCAGAGCTGGTTTAGTGAACCGTCAGATCCGGTAGCGCTACCGGTCGCCACC
ATGGTGAGCAAGGGCGAGGAGCTGTTCACCGGGGTGGTGCCCATCCTGGTCGAGCTGGACGG
CGACGTAAACGGCCACAAGTTCAGCGTGTCCGGCGAGGGCGAGGGCGATGCCACCTACGGCA
AGCTGACCCTGAAGTTCATCTGCACCACCGGCAAGCTGCCCGTGCCCTGGCCCACCCTCGTG
ACCACCCTGACCTACGGCGTGCAGTGCTTCAGCCGCTACCCCGACCACATGAAGCAGCACGA
CTTCTTCAAGTCCGCCATGCCCGAAGGCTACGTCCAGGAGCGCACCATCTTCTTCAAGGACG
ACGGCAACTACAAGACCCGCGCCGAGGTGAAGTTCGAGGGCGACACCCTGGTGAACCGCATC
GAGCTGAAGGGCATCGACTTCAAGGAGGACGGCAACATCCTGGGGCACAAGCTGGAGTACAA
CTACAACAGCCACAACGTCTATATCATGGCCGACAAGCAGAAGAACGGCATCAAGGTGAACT
TCAAGATCCGCCACAACATCGAGGACGGCAGCGTGCAGCTCGCCGACCACTACCAGCAGAAC
ACCCCCATCGGCGACGGCCCCGTGCTGCTGCCCGACAACCACTACCTGAGC*A*CCCAGTCCGC
CCTGAGCAAAGACCCCAACGAGAAGCGCGATCACATGGTCCTGCTGGAGTTCGTGACCGCCG
CCGGG*A*TCACTCTCGGCATGGACGAGCTGTACAAGTGAGGATCATCAAGCTGGGCTGCAGGA
ATTCGATATCAAGCTTATCGATAATCAACCTCTGGATTACAAAATTTGTGAAAGATTGACTG
GTATTCTTAACTATGTTGCTCCTTTTACGCTATGTGGATACGCTGCTTTAATGCCTTTGTAT
CATGCTATTGCTTCCCGTATGGCTTTCATTT*T*CTCCTCCTTGTATAAATCCTGGTTGCTGTC
TCTTTATGAGGAGTTGTGGCCCGTTGTCAGGCAACGTGGCGTGGTGTGCACTGTGTTTGCTG
ACGCAACCCCCACTGGTTGGGGCATTGCCACCACCTGTCAGCTCCTTTCCGGGACTTTCGCT
TTCCCCCTCCCTATTGCCACGGCGGAACTCATCGCCGCCTGCCTTGCCCG

Supporting text S3: Sequencing result for 5' RACE PCR result of D1 antisense lncRNAs

The sequence underlined is the 5' adaptor.
The sequence in red is the vector sequence.
The starting site for the antisense lncRNA is represented by the lowercase "c", which is located at about 743bps from the 3' terminal of the integrated vector.

<u>CGACTGGAGCACGAGGACACTGACATGGACTGAAGGAGTAGAAA</u>cTTTAACTCAATATAGC
ATTTATCATCATCTCTTCCCTCCCACTTGTTAAAGAACTAGCTTGTTCCAAATATGGCATTA
TCTTTTCCAAGTTCTAGGGTTTAGGCATGACATAATTCCTTTAAAGAATTATTTTTCTTGTC
ATGACTGACAAGCATGACTTAAATGGAGATAAACAGGCTACTGAAAAACATTTGGGAATTTT
TCTGATTACAACTGCTCTTATTTTTATTTTTTTTTTTGAGACAGAGTCTCGCTCTGTCGC
CCAGGCTGGAGTGCAGTGGCGCAATCTCAACTCACTGCAACCTTCGCCTCCCAGGTTCAAGC
GATTCTCCTGCCTCAGCTGCCTGAGTAGCTGGGACTACAGGTACCCACCACCAGGCCTAAAT
AATTTTTGTATTGTTAGTAGAGACGGGTTTCACCATGTTGGCCAGGCTGGTGTCAAACTCCT
GACCTCAGGTGATCCACCTCGGCCTCCCAAAGTGCTGGGATTACAGGCGTGAGCCACCATGC
CCAGTGTACAATTGCTCTTTTATGATGAGAGAAAAACAATGTATAGCTTTAAAATGGGGAAT
CCACATAAAACTATCTTCCCTTTGAACTCCAGTACAGGGGCTTTTGCCAATTAAACAAGTCA
CCTCCACCTGAGGAAAGAGGCAGACGCTGCCTGCCCAGGATGCTGTAGAAGGGATTCCTTCC
ATGGATAGAAAGTTTCTCTGCATAACTTTTGTAAAGGTCCTTTTGCTAGAGATTTTCCACAC
TGACTAAAAGGGTCTGAGGGATCTCTAGTTACCAGAGTCACACAACAGACGGGCACACACTA
CTTGAAGCACTCAAGGCAAGCTTTATTGAGGCTTAAGCAGTGGGTTCCCTAGTTAGCCAGAG
AGCTCCCAGGCTCA

Supporting text S4: The sequencing result for the antisense RNAs in E3 cells detected by 3' RACE PCR.

The red underlined letters are the detected termini of antisense RNAs that are transcribed into the integration sites. The underlined sequences are the primer binding sites.

TACGGTAAACTGCCCACTTGGCAGTACATCAAGTGTATCATATGCCAAGTACGCCCCCTATT
GACGTCAATGACGGTAAATGGCCCGCCTGGCATTATGCCCAGTACATGACCTTATGGGACTT
TCCTACTTGGCAGTACATCTACGTATTAGTCATCGCTATTACCATGGTGATGCGGTTTTGGC
AGTACATCAATGGGCGTGGATAGCGGTTTGACTCACGGGGATTTCCAAGTCTCCACCCCATT
GACGTCAATGGGAGTTTGTTTTGGCACCAAAATCAACGGGACTTTCCAAAATGTCGTAACAA
CTCCGCCCCATTGACGCAAATGGGCGGTAGGCGTGTACGGTGGGAGGTCTATATAAGCAGAG
CTGGTTTAGTGAACCGTCAGATCCGGTAGCGCTACCGGTCGCCACCATGGTGAGCAAGGGCG
AGGAGCTGTTCACCGGGGTGGTGCCCATCCTGGTCGAGCTGGACGGCGACGTAAACGGCCAC
AAGTTCAGCGTGTCCGGCGAGGGCGAGGGCGATGCCACCTACGGCAAGCTGACCCTGAAGTT
CATCTGCACCACCGGCAAGCTGCCCGTGCCCTGGCCCACCCTCGTGACCACCCTGACCTACG
GCGTGCAGTGCTTCAGCCGCTACCCCGACCACATGAAGCAGCACGACTTCTTCAAGTCCGCC
ATGCCCGAAGGCTACGTCCAGGAGCGCACCATCTTCTTCAAGGACGACGGCAACTACAAGAC
CCGCGCCGAGGTGAAGTTCGAGGGCGACACCCTGGTGAACCGCATCGAGCTGAAGGGCATCG
ACTTCAAGGAGGACGGCAACATCCTGGGGCACAAGCTGGAGTACAACTACAACAGCCACAAC
GTCTATATCATGGCCGACAAGCAGAAGAACGGCATCAAGGTGAACTTCAAGATCCGCCACAA
CATCGAGGACGGCAGCGTGCAGCTCGCCGACCACTACCAGCAGAACACCCCCATCGGCGACG
GCCCCGTGCTGCTGCCCGACAACCACTACCTGAGCACCCAGTCCGCCCTGAGCAAAGACCCC
AACGAGAAGCGCGATCACATGGTCCTGCTGGAGTTCGTGACCGCCGCCGGGATCACTCTCGG
CATGGACGAGCTGTACAAGTGAGGATCATCAAGCTGGGCTGCAGGAATTCGATATCAAGCTT
ATCGATAATCAACCTCTGGATTACAAAATTTGTGAAAGATTGACTGGTATTCTTAACTATGT
TGCTCCTTTTACGCTATGTGGATACGCTGCTTTAATGCCTTTGTATCATGCTATTGCTTCCC
GTATGGCTTTCATTTTCTCCTCCTTGTATAAATCCTGGTTGCTGTCTCTTTATGAGGAGTTG
TGGCCCGTTGTCAGGCAACGTGGCGTGGTGTGCACTGTGTTTGCTGACGCAACCCCCACTGG
TTGGGGCATTGCCACCACCTGTCAGCTCCTTTCCGGGACTTTCGCTTTCCCCCTCCCTATTG
CCACGGCGGAACTCATCGCCGCCTGCCTTGCCCG

Supporting text S5: Sequencing result for the antisense RNAs in PA5 cells detected by 5' RACE

The sequence underlined is the 5' adaptor.
The sequence in red is the vector sequence.
The starting site for the antisense lncRNA is represented by the lowercase "t", which is located at about 273 bp from the 3' terminal of the integrated vector.

T<u>GGACACTGACATGGACTGAAGGAGTAGAAA</u>tGACTACTAAGCATTTCTATCCAATATTCAA
TAGATAGGTTTATTCAAACTTTATGGCTTTCATGACTCCCTACTCATATTCTGTATGAATAA
TAAAAAATAATAATTTCCAACCCAGTTTTTCATGAGAGATAGTGTGGCATGATGTAAAAAGC
CCTGGCTTTGGAGTAAGAAATAACTGGATTCAACTTATGGTCCTCTTAGGCTGTGTGACTTG
AGGCTAGGTATATGAGCTCTCTAAACTTCAGTTTACTCAGCTAAAAAGTAGGTTATTGCTAG
AGATTTTCCACACTGACTAAAAGGGTCTGAGGGATCTCTAGTTACCAGAGTCACACAACAGA
CGGGCACACACTACTTGAAGCACTCAAGGCAAGCTTTATTGAGGCTTAAGCAGTGGGTTCCC
TAGTTAGCCAGAGAGCTCCCAGGCTCA

Figure S1

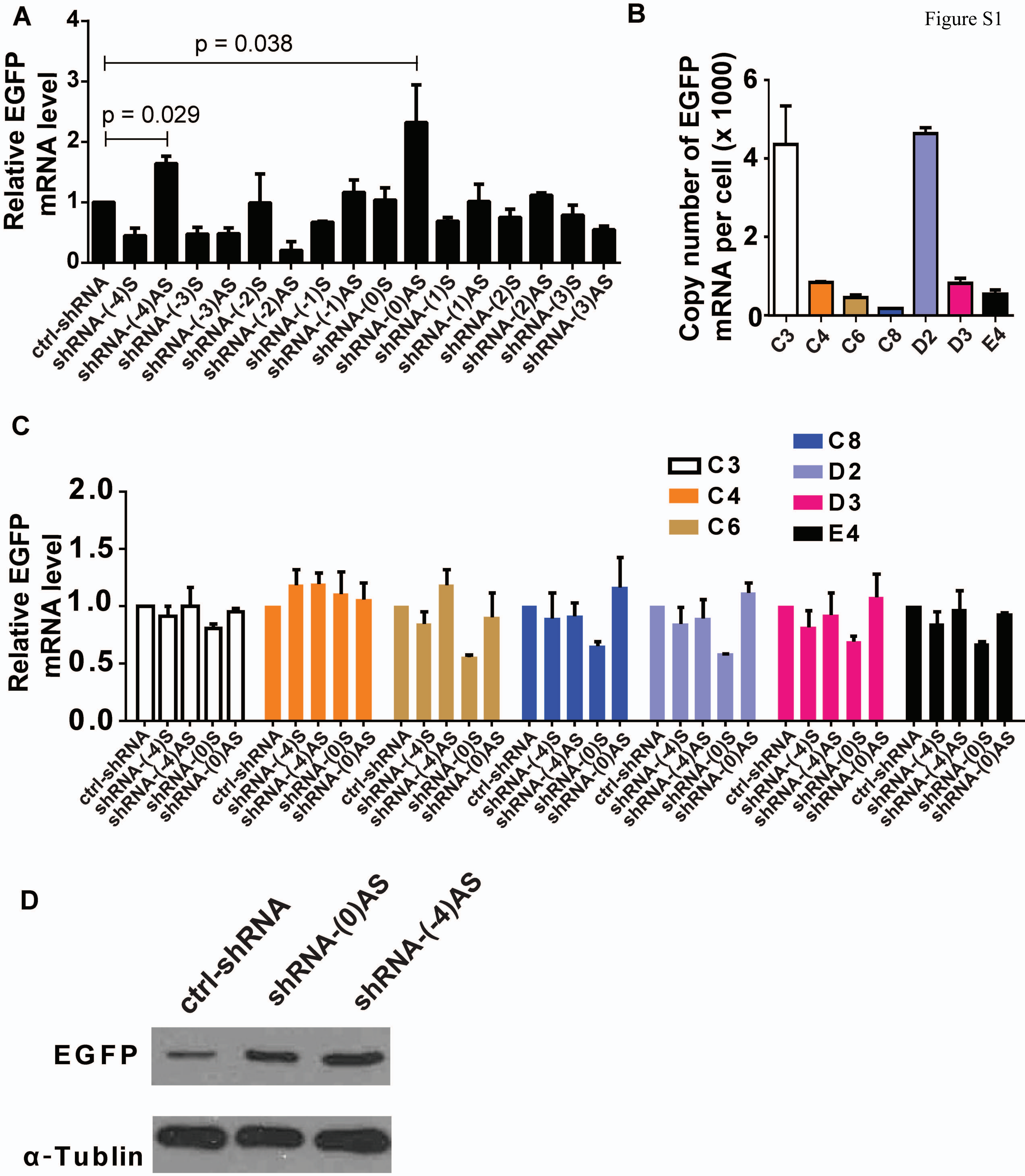
A
Relative EGFP mRNA level
p = 0.038
p = 0.029
ctrl-shRNA
shRNA-(-4)S
shRNA-(-4)AS
shRNA-(-3)S
shRNA-(-3)AS
shRNA-(-2)S
shRNA-(-2)AS
shRNA-(-1)S
shRNA-(-1)AS
shRNA-(0)S
shRNA-(0)AS
shRNA-(1)S
shRNA-(1)AS
shRNA-(2)S
shRNA-(2)AS
shRNA-(3)S
shRNA-(3)AS
B
Copy number of EGFP mRNA per cell (x 1000)
C3
C4
C6
C8
D2
D3
E4
C
Relative EGFP mRNA level
C3
C4
C6
C8
D2
D3
E4
ctrl-shRNA
shRNA-(-4)S
shRNA-(-4)AS
shRNA-(0)S
shRNA-(0)AS
D
ctrl-shRNA
shRNA-(0)AS
shRNA-(-4)AS
EGFP
α-Tublin

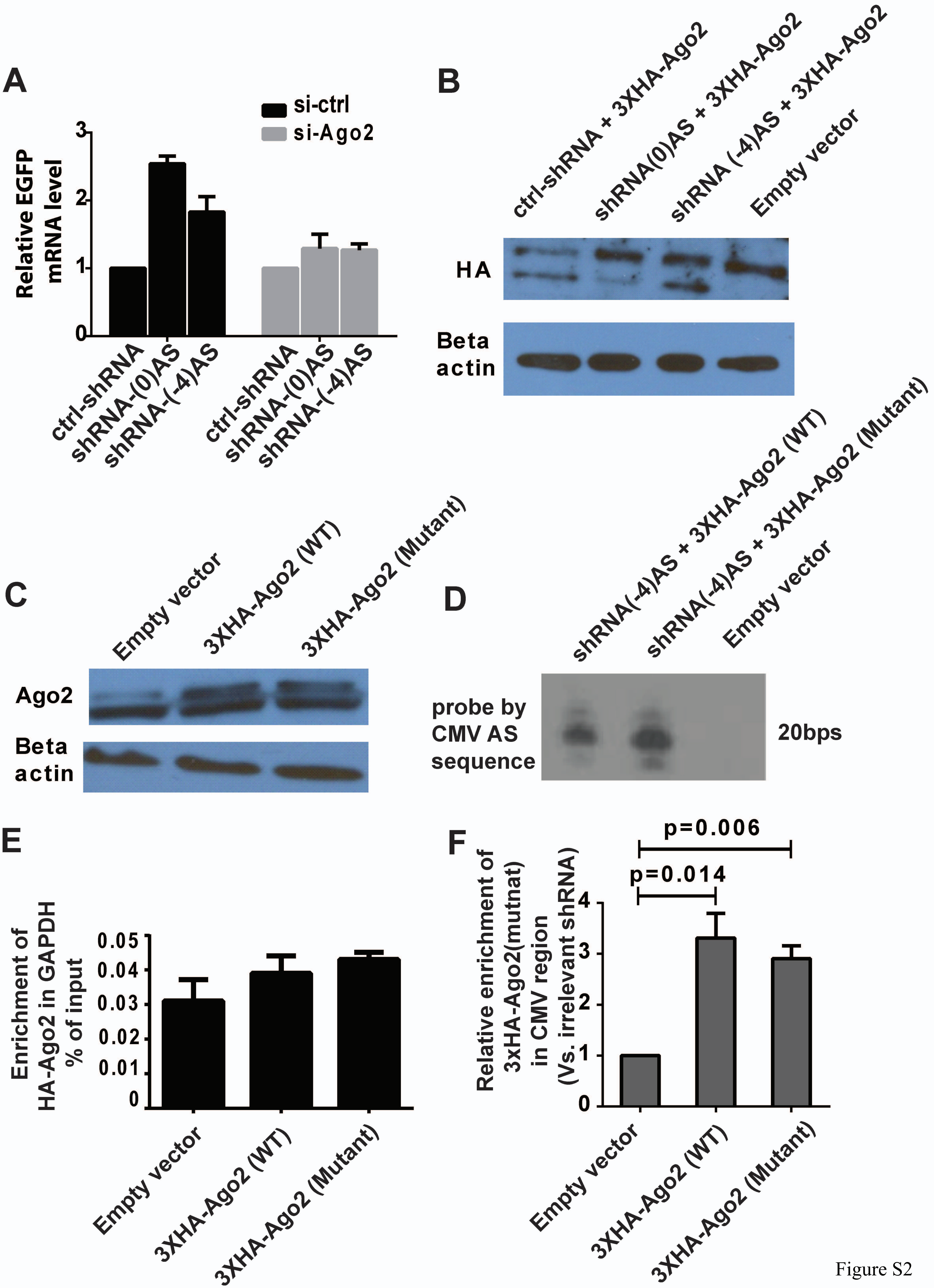
A
si-ctrl
si-Ago2
Relative EGFP mRNA level
0
1
2
3
ctrl-shRNA
shRNA-(0)AS
shRNA-(-4)AS
ctrl-shRNA
shRNA-(0)AS
shRNA-(-4)AS
B
ctrl-shRNA + 3XHA-Ago2
shRNA(0)AS + 3XHA-Ago2
shRNA (-4)AS + 3XHA-Ago2
Empty vector
HA
Beta actin
C
Empty vector
3XHA-Ago2 (WT)
3XHA-Ago2 (Mutant)
Ago2
Beta actin
D
shRNA(-4)AS + 3XHA-Ago2 (WT)
shRNA(-4)AS + 3XHA-Ago2 (Mutant)
Empty vector
probe by CMV AS sequence
20bps
E
Enrichment of HA-Ago2 in GAPDH % of input
0
0.01
0.02
0.03
0.04
0.05
Empty vector
3XHA-Ago2 (WT)
3XHA-Ago2 (Mutant)
F
Relative enrichment of 3xHA-Ago2(mutnat) in CMV region (Vs. irrelevant shRNA)
p=0.006
p=0.014
0
1
2
3
4
Empty vector
3XHA-Ago2 (WT)
3XHA-Ago2 (Mutant)

Figure S2

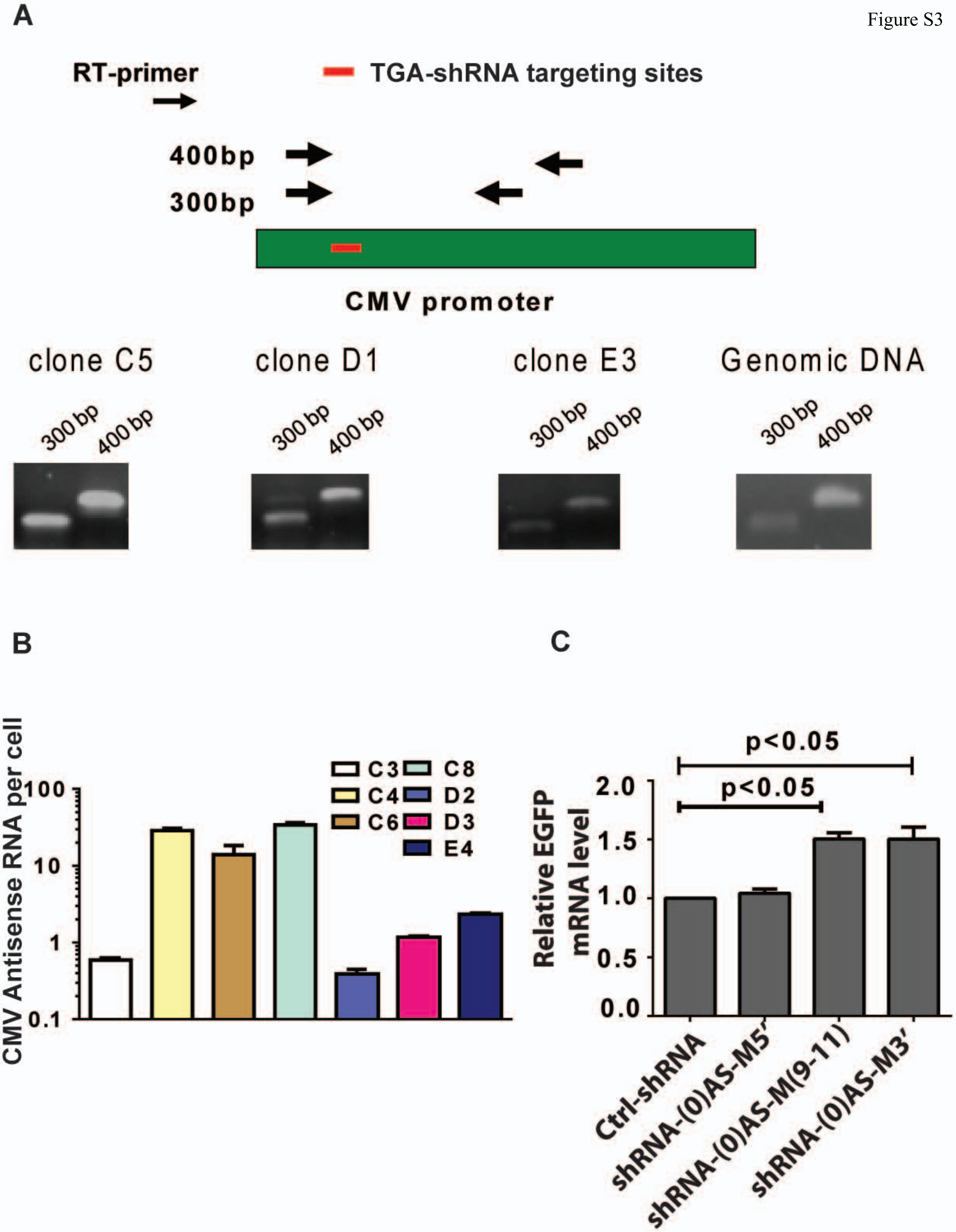
A
RT-primer
TGA-shRNA targeting sites
400bp
300bp
CMV promoter
clone C5
clone D1
clone E3
Genomic DNA
300 bp
400 bp
B
copy number of
CMV Antisense RNA per cell
100
10
1
0.1
C3
C4
C6
C8
D2
D3
E4
C
Relative EGFP
mRNA level
2.0
1.5
1.0
0.5
0.0
p<0.05
p<0.05
Ctrl-shRNA
shRNA-(0)AS-M5′
shRNA-(0)AS-M(9-11)
shRNA-(0)AS-M3′

Figure S4

A

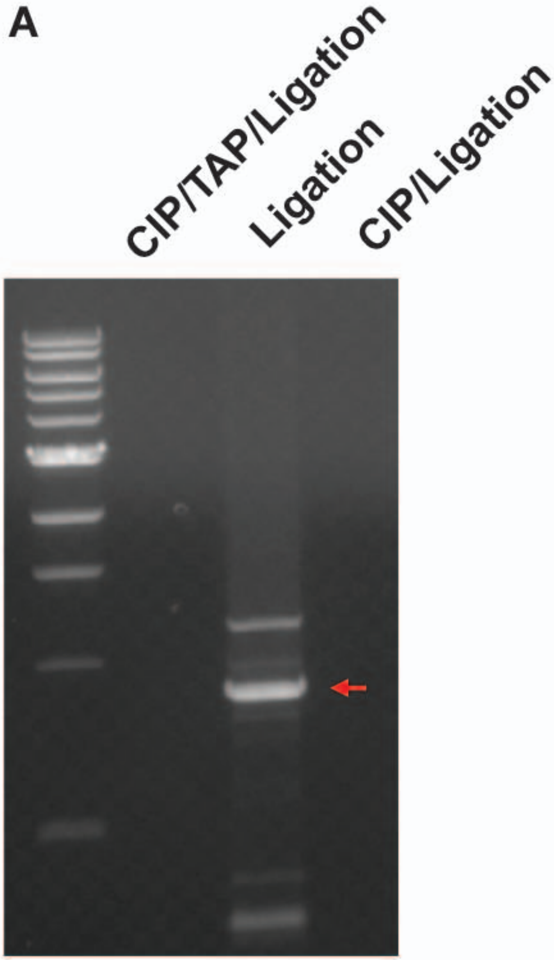
CIP/TAP/Ligation
Ligation
CIP/Ligation

B

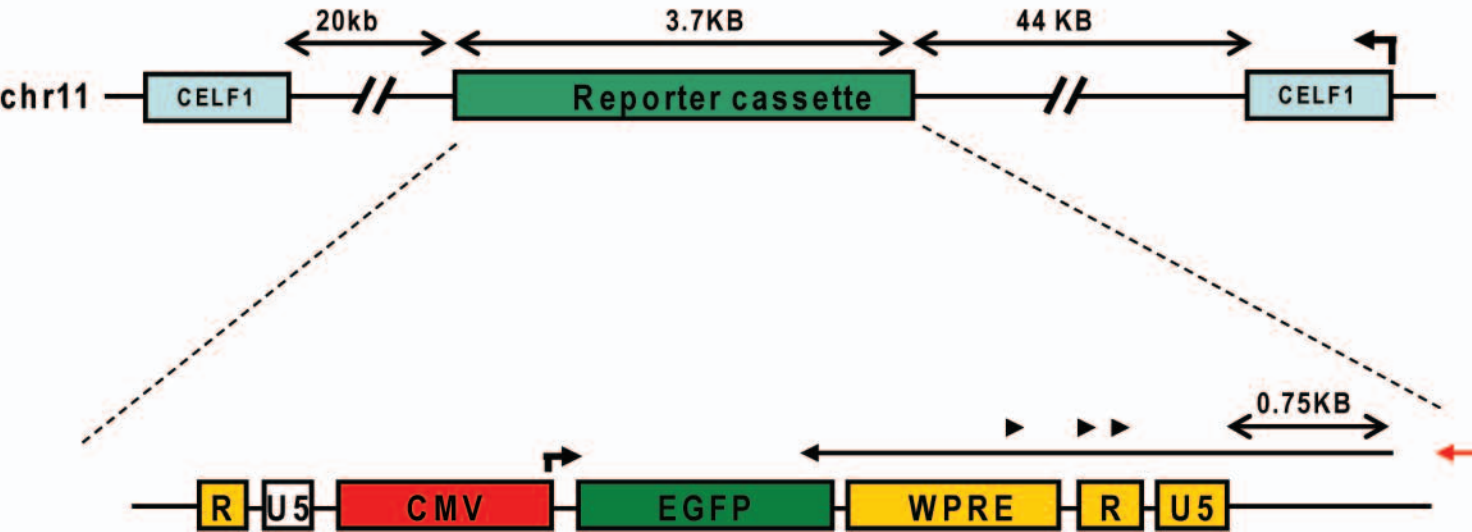
20kb
3.7KB
44 KB
chr11
CELF1
Reporter cassette
CELF1
0.75KB
R
U5
CMV
EGFP
WPRE
R
U5

Figure S5

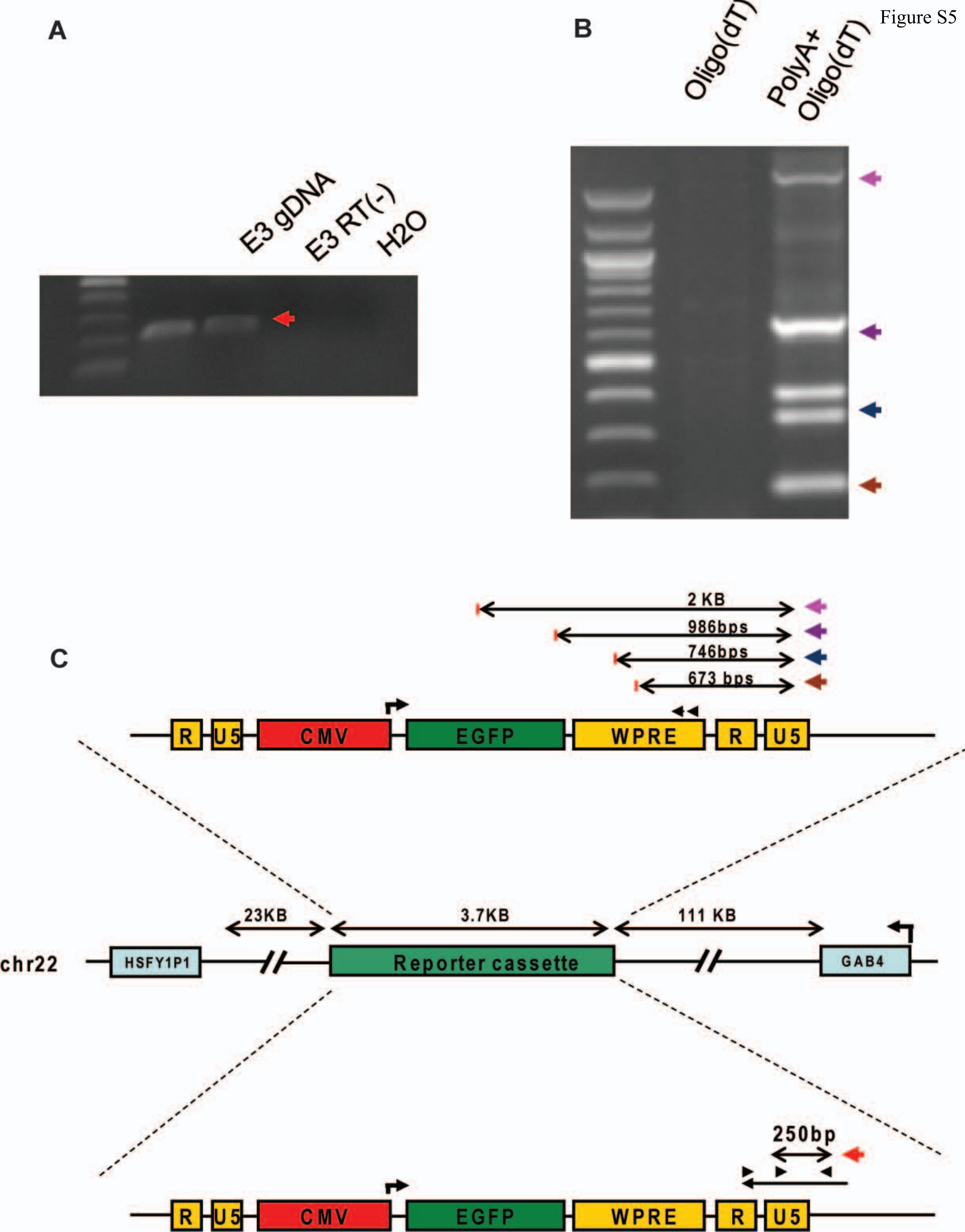
A
E3 gDNA
E3 RT(-)
H2O
B
Oligo(dT)
PolyA+ Oligo(dT)
C
2 KB
986bps
746bps
673 bps
R
U5
CMV
EGFP
WPRE
R
U5
23KB
3.7KB
111 KB
chr22
HSFY1P1
Reporter cassette
GAB4
250bp
R
U5
CMV
EGFP
WPRE
R
U5

Figure S6

A

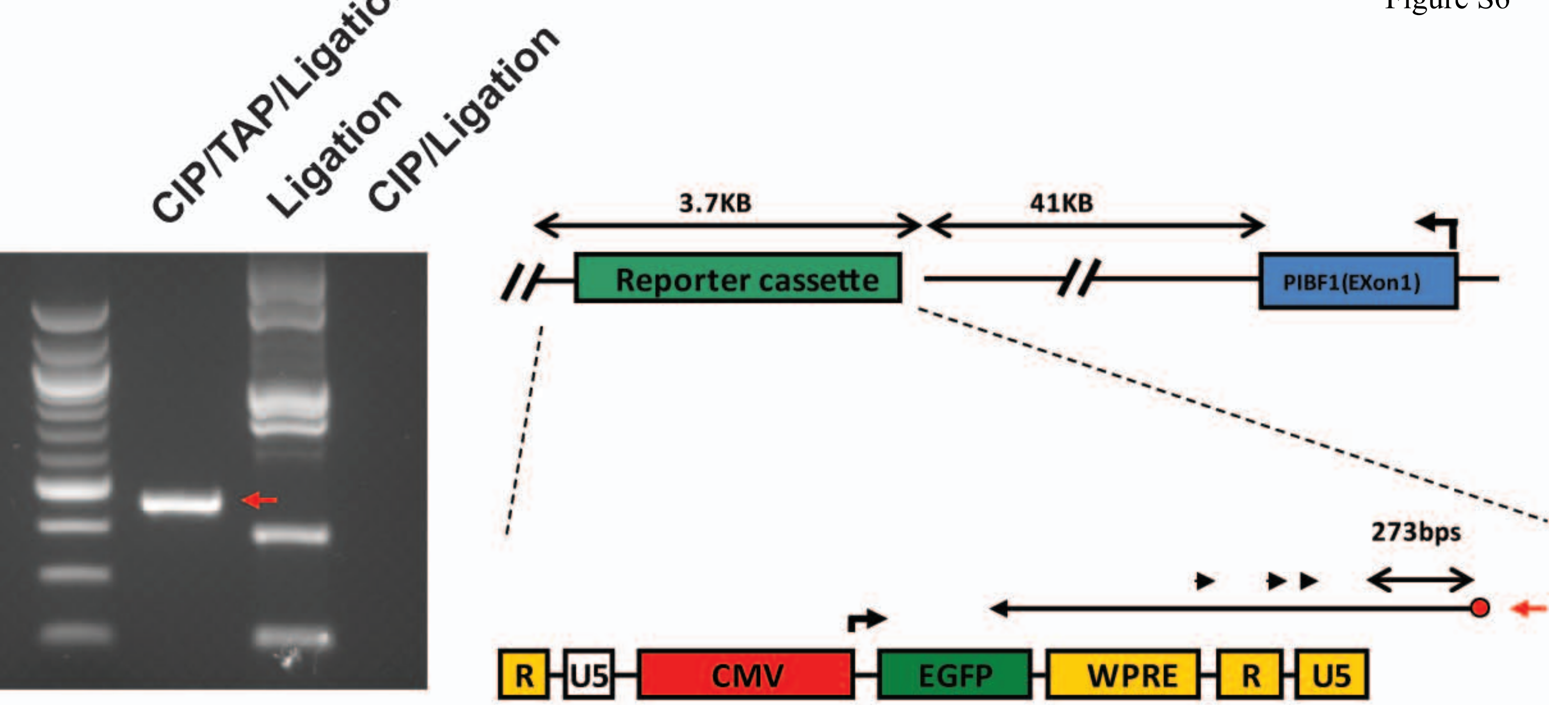
CIP/TAP/Ligation
Ligation
CIP/Ligation
3.7KB
41KB
Reporter cassette
PIBF1(EXon1)
273bps
R
U5
CMV
EGFP
WPRE
R
U5

B

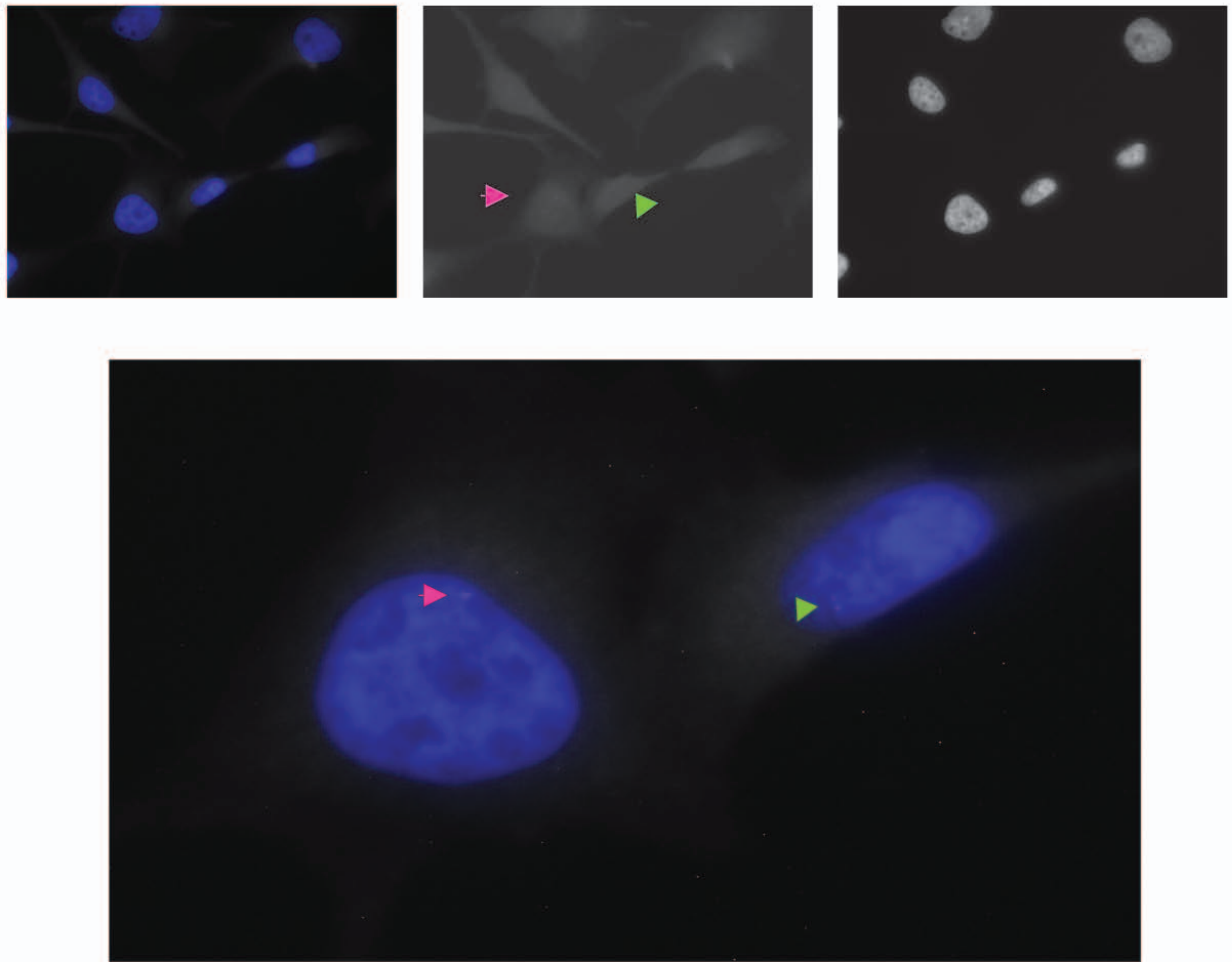

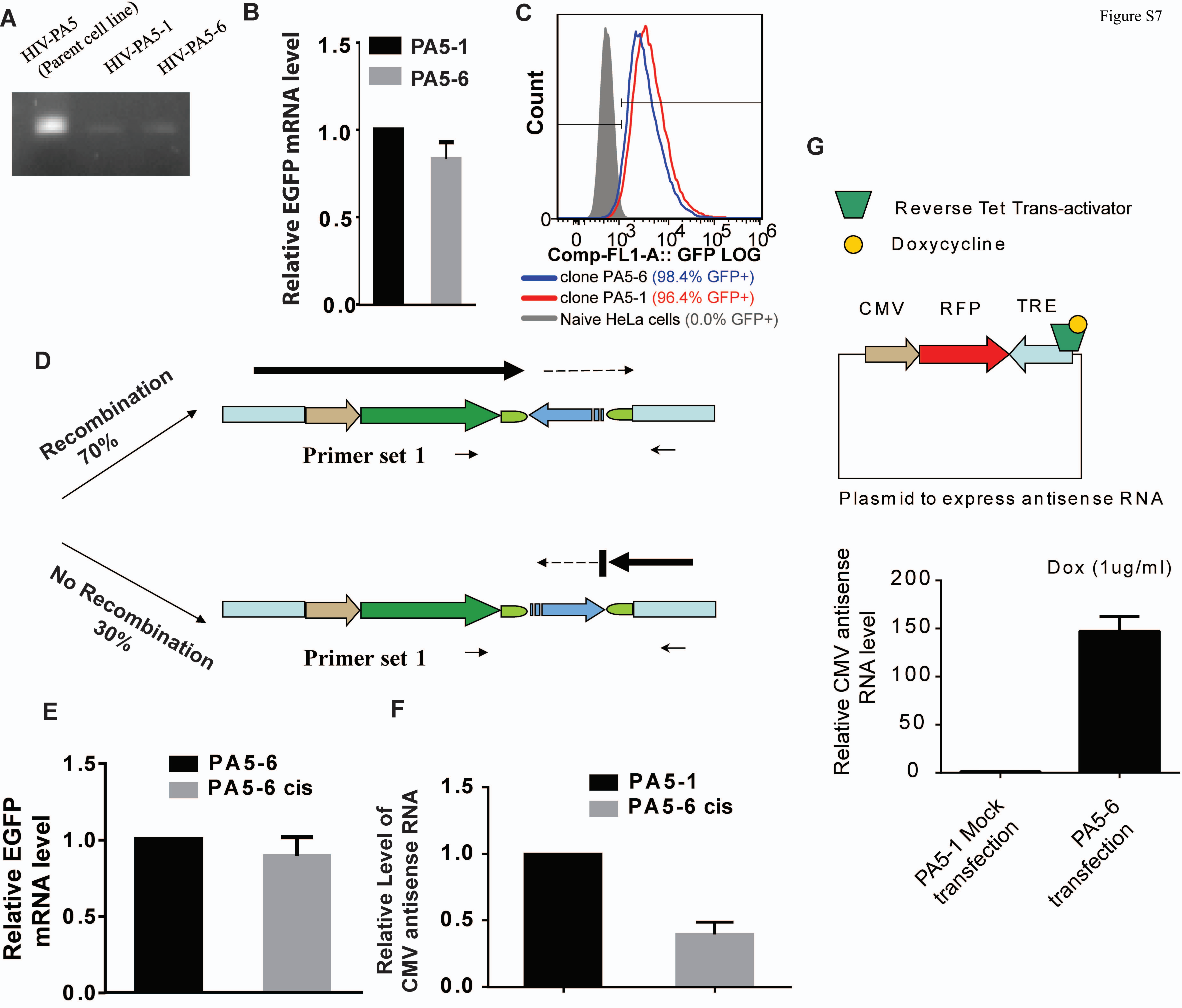
A
HIV-PA5 (Parent cell line)
HIV-PA5-1
HIV-PA5-6
B
Relative EGFP mRNA level
PA5-1
PA5-6
1.5
1.0
0.5
0.0
C
Count
Comp-FL1-A:: GFP LOG
clone PA5-6 (98.4% GFP+)
clone PA5-1 (96.4% GFP+)
Naive HeLa cells (0.0% GFP+)
D
Recombination 70%
No Recombination 30%
Primer set 1
Primer set 1
E
Relative EGFP mRNA level
PA5-6
PA5-6 cis
F
Relative Level of CMV antisense RNA
PA5-1
PA5-6 cis
G
Reverse Tet Trans-activator
Doxycycline
CMV
RFP
TRE
Plasmid to express antisense RNA
Dox (1ug/ml)
Relative CMV antisense RNA level
PA5-1 Mock transfection
PA5-6 transfection
